# Antibacterial and antivirulence activities of abafungin against *Clostridioides difficile*: *in vitro* and *in vivo* studies

**DOI:** 10.1128/aac.00598-26

**Published:** 2026-08-13

**Authors:** Ahmed A. Abouelkhair, Nader S. Abutaleb, Sahar Abdelrazek, Mohamed N. Seleem

**Affiliations:** 1 Department of Biomedical Sciences and Pathobiology, Virginia-Maryland College of Veterinary Medicine, Virginia Polytechnic Institute and State University 1757, Blacksburg, Virginia, USA; 2 Department of Bacteriology, Mycology, and Immunology, Faculty of Veterinary Medicine, University of Sadat City 392053, Sadat City, Menofia, Egypt; 3 Department of Microbiology and Immunology, Faculty of Pharmacy, Zagazig University 68799, Zagazig, Al-Sharqia, Egypt; 4 Department of Plant Pathology, Faculty of Agriculture, Assiut University, Assiut, Egypt; 5 Center for One Health Research, Virginia Polytechnic Institute and State University 1757, Blacksburg, Virginia, USA; Columbia University Irving Medical Center, New York, New York, USA

**Keywords:** *Clostridioides difficile*, spore germination and outgrowth, toxin inhibition, repurposing, RNA_seq, metal homeostasis, CDI mouse model

## Abstract

*Clostridioides difficile* infection (CDI) remains a major cause of healthcare-associated diarrhea worldwide, and is classified as an urgent public health threat by the Centers for Disease Control and Prevention. Current frontline therapies, including vancomycin and fidaxomicin, are associated with disruption of the intestinal microbiota, recurrence, and emerging reduced susceptibility, underscoring the need for additional therapeutic strategies. Using a drug-repurposing approach, we identified the antifungal agent abafungin as a potent inhibitor of *C. difficile*. Abafungin is an arylguanidine antifungal with reported sporicidal activity against fungal pathogens, a property of particular relevance to CDI, given the central role of spores in disease persistence and recurrence. Herein, abafungin exhibited potent *in vitro* activity against a diverse panel of clinical *C. difficile* isolates, inhibiting 50% (MIC_5₀_) and 90% (MIC_₉₀_) of the tested strains at the concentrations of 0.25 μg/mL and 0.5 μg/mL, respectively, while demonstrating minimal activity against representative gut commensal bacteria. At sub-inhibitory concentrations, abafungin significantly reduced toxin production (31.8%), decreased spore formation (~4-log₁₀ reduction), and inhibited spore germination. Abafungin showed favorable gastrointestinal stability and demonstrated synergistic activity with vancomycin and metronidazole. Transcriptomic analysis revealed that abafungin disrupts metal ion transport and intracellular metal homeostasis in *C. difficile*, a mechanism supported by the antagonistic effect of Mg²^+^ supplementation on its antibacterial activity. These observations suggest a potential association between abafungin activity and metal-responsive cellular pathways. In a murine model of recurrent CDI, a combination therapy of abafungin and vancomycin significantly improved survival (83.3%) compared with vancomycin monotherapy (16.6%). Collectively, these findings establish abafungin as a potential anti*-C*. *difficile* inhibitor that suppresses *C. difficile* growth and virulence-associated processes and enhances the therapeutic efficacy of vancomycin *in vivo*. Also, these observations provide a basis for further studies exploring abafungin as a useful lead scaffold for anti*-C*. *difficile* drug discovery.

## INTRODUCTION

*Clostridioides difficile* infection (CDI) is the most common healthcare-associated infection in the United States and remains an urgent global public health threat. Individuals with recent exposure to broad-spectrum antibiotics are at increased risk of CDI due to disruption of the protective gut microbiota, which compromises colonization resistance and facilitates *C. difficile* establishment and proliferation (1, 2). CDI causes a spectrum of symptoms ranging from antibiotic-associated diarrhea to life-threatening fulminant colitis, toxic megacolon, and sepsis. In 2017, the Centers for Disease Control and Prevention (CDC) reported 223,900 CDI cases among hospitalized patients and approximately 12,800 associated deaths in the USA, with total healthcare costs exceeding $1 billion annually (3). Recently, the 2024 report from the CDC Emerging Infections Program (EIP) estimated a CDI incidence rate of 116.1 cases per 100,000 individuals across EIP surveillance sites. Notably, more than 50% of reported CDI cases were classified as community-acquired, with the majority of these cases associated with recent outpatient healthcare exposures (4, 5). Hence, due to its significant morbidity, mortality, and economic burden, CDI is categorized by the CDC as an urgent threat (6).

The primary drivers of CDI pathogenesis are toxin A (TcdA) and toxin B (TcdB), which enter colonic epithelial cells and inactivate Rho GTPases, resulting in cytoskeletal breakdown, epithelial barrier damage, and severe mucosal inflammation (7). In addition, *C. difficile* produces highly resilient spores that persist on surfaces for extended durations, up to several months, facilitating transmission and recurrence. Upon exposure to bile acids and other germination signals within the gastrointestinal tract, *C. difficile* spores transition into toxin-producing vegetative cells. Consequently, spore germination and subsequent outgrowth represent critical processes contributing to disease recurrence and relapse (8, 9).

Clinically, metronidazole (MTZ) was previously recommended as a first-line therapy for adult CDI; however, its use has declined due to increasing rates of treatment failure. Current guidelines recommend MTZ only for non-severe CDI in situations where access to the two approved anti-*C*. *difficile* drugs, vancomycin (VAN) or fidaxomicin (FDX), is limited (10). Even though VAN exhibits strong activity against CDI, its use is associated with some limitations, including disruption of the intestinal microbiota, treatment failure in some cases, and relatively high recurrence rates (~35%) (11). FDX offers advantages over VAN, including improved preservation of the intestinal microbiota and enhanced inhibition of spore formation and toxin production, which contribute to lower recurrence rates (~19.7%) (12–16). However, CDI recurrence remains a significant clinical challenge, and the broader implementation of FDX is limited by its high cost and reduced activity against certain hypervirulent *C. difficile* ribotypes (17, 18). Recently approved microbiome restoration therapies, such as fecal microbiota transplantation (FMT) products (Rebyota and Vowst), provide benefit in preventing recurrence (19), but are constrained by cost, safety concerns in immunocompromised populations, and uncertain long-term safety (20). Moreover, FMT products are not approved for treatment of acute CDI and for patients under 18 years (21). Collectively, these limitations underscore the continued need to develop new therapeutic strategies for the treatment of CDI.

To identify additional treatment options, drug repurposing has emerged as an efficient strategy that accelerates drug discovery by identifying new clinical applications for existing drugs with established pharmacological and safety profiles (22–24). Using the drug-repurposing approach, we identified abafungin as a potent inhibitor of *C. difficile* at comparable activity with the drug of choice. Abafungin is a broad-spectrum antifungal agent originally discovered by Bayer AG (Leverkusen, Germany) during screening efforts aimed at identifying H₂-receptor antagonists. Structurally, it belongs to the arylguanidine class and is distinct from conventional azole antifungals. Importantly, abafungin has been reported to exhibit sporicidal activity against fungal pathogens (25, 26), a property that may be particularly advantageous for targeting *C. difficile*, where spores play a central role in disease persistence and recurrence (26–28). In addition, abafungin possesses fungicidal activity against *Candida* species and other yeasts (26). This dual activity against fungal organisms and *C. difficile* may be advantageous in the context of fungal–bacterial interactions within the gut microbiota, where antibiotic-induced fungal expansion has been shown to exacerbate intestinal inflammation and promote CDI in experimental models (29–31). Collectively, these properties provided a strong rationale for further investigation of abafungin as a potential therapeutic candidate for CDI.

In the present study, we report for the first time the antibacterial activity of abafungin against *C. difficile*. We evaluated its activity against a panel of clinical isolates and investigated its effects on key virulence traits, including toxin production, spore formation, and spore germination. In addition, we assessed its stability under simulated gastrointestinal conditions, selectivity toward representative commensal gut bacteria, and cytotoxicity in mammalian cells. We further examined its interactions with existing anti-CDI therapeutics and investigated potential cellular pathways associated with abafungin activity to gain insights into its possible mechanism of action. Finally, the therapeutic efficacy of abafungin was evaluated in a murine model of recurrent *C. difficile* infection to determine whether combination therapy with VAN could enhance treatment outcomes and reduce relapse.

## MATERIALS AND METHODS

### Strains, drugs, chemicals, and media

*C. difficile* and gut microbiota isolates used in this study (Tables S1 and S2) were obtained from the Biodefense and Emerging Infections Research Resources Repository (BEI Resources), the American Type Culture Collection (ATCC), and the CDC. Drugs were purchased commercially: abafungin (TCI America), FDX (Biosynth Carbosynth), metronidazole (Alfa Aesar), and vancomycin (Gold Biotechnology). Gentamicin and kanamycin were purchased from Chem Impex, and clindamycin was purchased from TCI America.

Chemicals and media were purchased from different vendors: brain heart infusion (BHI) broth and de Man-Rogosa-Sharpe (MRS) broth (Becton, Dickinson, and Company); yeast extract (Fisher Scientific); Eagle’s Minimum Essential Medium (MEM) (ATCC); l-cysteine (Alfa Aesar); glycine, vitamin K, hemin, and magnesium chloride (Sigma-Aldrich); taurocholic acid sodium salt hydrate (TA) (Chem Impex); MTS3-(4,5-dimethylthiazol-2-yl)-5-(3-carboxymethoxyphenyl)-2-(4-sulfophenyl)-2H-tetrazolium); and phosphate-buffered saline (PBS) (Corning).

### Determination of the minimum inhibitory concentrations (MICs)

Minimum inhibitory concentrations (MICs) of abafungin against *C. difficile* strains and common gut bacteria were determined as described previously (32–35). In triplicates, abafungin, VAN, and FDX were serially diluted in either BHI-supplemented (BHIS) broth for *C. difficile*, *Bacteroides*, and *Bifidobacterium* strains, while MRS broth was used for *Lactobacillus* strains. A bacterial solution equivalent to 0.5 McFarland was prepared and diluted to a concentration of ~5 × 10^5^ CFU/mL and added to plates. Plates were incubated under anaerobic conditions for 48 h (for *C. difficile*, *Bacteroides*, and *Bifidobacterium* strains) or in the presence of 5% CO_2_ for 48 h (for *Lactobacillus* strains). The MIC of each antibiotic was determined as the lowest concentration that inhibited bacterial growth, as detected by visual inspection of the plates (36).

### Time-kill kinetics

A time-kill assay was performed to evaluate killing kinetics of abafungin against *C. difficile*, as previously described (37–39). *C. difficile* ATCC 43255 (ribotype 087) in the logarithmic phase was diluted to ~10^6^ CFU/mL and treated in triplicates with either abafungin (at 1×, 3×, and 5× MIC), VAN, or FDX (at 5× MIC) in pre-reduced BHIS broth. DMSO served as a growth control. Aliquots were taken from each treatment at 0, 2, 4, 8, 12, and 24 h, diluted, and plated onto pre-reduced BHIS agar plates.

### Toxin inhibition assay

To investigate abafungin’s effect on *C. difficile* toxin production, abafungin, VAN, FDX, or MTZ were added at 0.25× MIC to logarithmic cultures of *C. difficile* ATCC 43255 (~10^6^ CFU/mL) as previously described (39–41), followed by a 6-h incubation under anaerobic conditions. Culture supernatants were collected, filtered (0.22 µm), and toxin A (TcdA) and toxin B (TcdB) concentrations were quantified using a commercial ELISA kit (tgc BIOMICS) according to the manufacturer’s instructions (42, 43). Absorbance at 450/620 nm was measured using a BioTek Synergy H1 Hybrid reader. Toxin levels were normalized to viable bacterial counts to control for effects related to growth inhibition.

### Spore inhibition assay

We determined the inhibitory activity of abafungin against *C. difficile* spore formation as previously described (14). Log-phase cultures of *C. difficile* were diluted in BHIS to an initial density of 10^6^ CFU/mL and aliquoted into microcentrifuge tubes (triplicates). Abafungin, VAN, FDX, and MTZ were added at 0.25 and 0.5× MIC, followed by anaerobic incubation at 37°C for 6 days. To count the total bacterial counts (vegetative bacteria plus spores), an aliquot from each tube was diluted and plated on pre-reduced BHIS agar plates supplemented with 0.1% taurocholic acid. The remaining culture was heated (at 65°C for 30 min) to eliminate vegetative cells, centrifuged (4°C), resuspended in PBS, held overnight at 4°C, and enumerated after serial dilution and plating on pre-reduced BHIS agar plates supplemented with 0.1% taurocholic acid. Differences between total counts and spore counts were used to calculate sporulation activity.

### Spore germination assay

*C. difficile* ATCC 43255 was cultured on pre-reduced BHIS agar plates and incubated anaerobically for 48 h at 37°C. The colonies were then scraped off and spread out on pre-reduced 70:30 agar plates (44). Spores were harvested by scraping, washed twice in PBS, and pelleted by centrifugation (3,000 rpm, 10 min). Suspensions were heat-treated (65°C, 40 min) to remove remaining vegetative cells and stored at 4°C (45). The effect of abafungin on *C. difficile* spore germination was monitored as previously described (46). Approximately 10^7^ spores were suspended in 50 mM Tris HCl (pH, 7.4) aerobically at 37°C with or without germinant (0.2% taurocholic acid [TA] and 50 mM glycine) and exposed to abafungin or control drugs (VAN, FDX, and MTZ) at 5× MIC. Germination kinetics were monitored for 180 min by measuring OD₆₀₀ in a BioTek Synergy H1 plate reader. A decrease in OD₆₀₀ indicated transition to vegetative cells, consistent with spore germination (47).

### Cell cytotoxicity assay

To evaluate the potential toxic effect of abafungin, a cytotoxicity assay was performed against cell lines as described previously (43, 48, 49). Human epithelial colorectal (Caco-2) and kidney (Vero) cell lines (ATCC) were cultured in MEM supplemented with 20% FBS (Caco-2) or 10% FBS (Vero) and 1% penicillin–streptomycin at 37°C in the presence of 5% CO₂. Cells were seeded into treated 96-well plates and exposed to abafungin (up to 64 µg/mL). DMSO served as a negative control. After 24 h, cell viability was quantified using an MTS/PMS assay with absorbance read at 490 nm. Percent viability was calculated relative to DMSO readings.

### Transcriptomic profiling (RNA sequencing)

#### Experimental design and RNA isolation

*C. difficile* ATCC 630 was grown anaerobically in pre-reduced BHIS broth to mid-log phase. Cultures (~5 × 10^6^ CFU/mL) were treated for 3 h with abafungin or VAN at 0.5× MIC or DMSO (vehicle control). Three independent biological replicates were performed for each treatment condition. Following treatment, RNA was stabilized by the addition of RNAprotect Bacteria Reagent (Qiagen) at 4× the culture volume, as previously described (50). Cells were enzymatically lysed with lysozyme (15 µg/mL) and proteinase K, followed by mechanical disruption using 0.5 mm glass beads in a TissueLyser for 5 min. Total RNA was extracted using the RNeasy Plus Kit (Qiagen), including genomic DNA removal with gDNA Eliminator spin columns and on-column DNase treatment. Final purification was performed using the RNeasy Mini kit (Qiagen). RNA purity and yield were assessed using a NanoDrop spectrophotometer (Thermo Fisher Scientific), while RNA integrity was evaluated using an Agilent 4200 TapeStation.

#### rRNA depletion, library preparation, and RNA sequencing

Total RNA was quantified using a Qubit 2.0 Fluorometer (Thermo Fisher Scientific). Ribosomal RNA was depleted using the Vazyme Ribo-Clean rRNA Depletion Kit (Bacteria), and sequencing libraries were prepared with the VAHTS Universal V8 RNA-seq Library Prep Kit for Illumina according to the manufacturers’ protocols. Briefly, rRNA-depleted RNA was fragmented (94°C, 15 min), followed by first- and second-strand cDNA synthesis, end repair, A-tailing, adapter ligation, index incorporation, and PCR amplification. Library quality and fragment size distribution were assessed using the Agilent 4200 TapeStation, and library concentrations were determined by Qubit and qPCR (KAPA Biosystems). Multiplexed libraries were sequenced on an Illumina NovaSeq platform (2 × 150 bp paired-end). Base calling was performed using NovaSeq Control Software, and BCL files were converted to FASTQ format using bcl2fastq v2.20, allowing one index mismatch.

#### Transcriptomic analysis

FastQ files generated by RNA sequencing were processed through the Virginia Tech Advanced Research Computing (ARC) infrastructure. Read quality was evaluated using FastQC (51), and adapter sequences and low-quality bases were removed using Cutadapt (52). Only high-quality reads were retained for downstream analyses. High-quality reads were then aligned to the *C. difficile* ATCC 630 reference genome (NCBI accession GCF_000009205.2_ASM920v2) using Bowtie2 (53). The resulting SAM alignment files were converted to BAM format using SAMtools (54), and gene-level read counts were generated using the featureCounts program from the Subread package (55), specifying coding sequence (CDS) features. The resulting CDS count matrix was used for count-based differential gene expression and statistical analysis among treatments using the DESeq2 R package (56). To assess sample reproducibility and overall transcriptomic variation, principal component analysis (PCA) and hierarchical clustering analyses were performed using variance-stabilized count data. Heat maps were generated using the pheatmap R package to visualize sample clustering and expression patterns of differentially expressed genes. Volcano plots were generated using the EnhancedVolcano R package (57), applying thresholds of an adjusted *P*-value *(*padj) <0.001 and |log₂ fold change| ≥ 2. Genes identified as significantly differentially expressed were subjected to functional enrichment analysis using ShinyGO (58). Enriched biological processes and pathways were ranked based on statistical significance and fold enrichment.

### Cation chelation interference assay

To investigate whether the inhibitory activity of abafungin relies on chelation of cations, MICs of abafungin against *C. difficile* were determined in *C. difficile* minimal medium (CDMM) supplemented with individual cations (Ca²^+^, Na^+^, Zn²^+^, Fe²^+^, Mn²^+^, K^+^, Mg²^+^; each at 3 mM) (59). Briefly, the MICs of abafungin and control antibiotics were determined against *C. difficile* strains in the presence of different cations (CaCl_2_, NaCl, ZnCl_2_, FeSO_4_, MnSo_4_, KCl, MgCl_2_), as described above.

### Time-kill kinetics of abafungin in presence of magnesium salt

To evaluate magnesium-dependent antagonism, log-phase cultures (~10⁶ CFU/mL) were treated with abafungin at 5× MIC alone or in the presence of MgCl₂ (5–30 mM). Cells without abafungin served as growth control. Aliquots from each treatment were taken after 0, 4, 8, and 12 h, diluted, and plated onto pre-reduced BHIS agar plates in order to determine the CFU counts.

### Checkerboard assay

The interactions between abafungin and either VAN or MTZ were evaluated via checkerboard assay, as reported previously (42, 45). In 96-well plates, 2-fold serial dilutions of abafungin were combined with either VAN (0.0039–2 μg/mL) or MTZ (0.0019–1μg/mL). Bacteria were added, and plates were incubated anaerobically for 48 h at 37°C. Fractional inhibitory concentration index (FICI) was derived using the formula:


$$FICI=\frac{MICabafunginincombination}{MICabafunginalone}+\frac{MICVANorMTZincombination}{MICVANorMTZalone}$$

To interpret the results, FICI ≤ 0.5 was considered synergy (SYN), and 0.5 §amp;lt; FICI §amp;lt; 1.25 was considered additive (ADD), whereas a value of >1.25 to 4 was considered indifference (IND), and FICI > 4 is antagonistic (ANT).

### Stability in gastrointestinal fluids

To ascertain the MICs of abafungin, VAN, and FDX in the presence of simulated gastric fluid (SGF) and simulated intestinal fluid (SIF), the broth microdilution test was performed. SGF (pH 1.2) and SIF (pH = 6.8) were prepared as previously described (60). In triplicates, abafungin, VAN, and FDX were incubated with SGF or SIF for 0, 4, or 24 h prior to MIC determination against *C. difficile* ATCC 43255 and ATCC BAA-1870 via broth microdilution, as described above.

### *In vivo* efficacy

Female 8-week-old C57BL/6 mice (Jackson Laboratories) were housed in sterile, individually ventilated cages with *ad libitum* access to food and water. CDI mouse model was performed as described previously (32, 61, 62). Mice received an antibiotic cocktail in drinking water for 5 days (kanamycin 0.4 mg/mL, gentamicin 35 mg/mL, colistin 850 U/mL, metronidazole 0.215 mg/mL, vancomycin 0.045 mg/mL, and sucrose 75 mg/mL), followed by 2 days of antibiotic-free water. Clindamycin (10 mg/kg, i.p.) was administered 24 h before challenge.

Mice were orally infected with 2.6 × 10⁵ spores of *C. difficile* ATCC 43255 via oral gavage using a ball-tipped metal feeder. Following infection, mice were randomly allocated into groups (*n* = 6) for treatment. Two-hours post-infection, mice were treated with abafungin (20 mg/kg) and VAN (10 mg/kg) once daily via oral gavage. Control groups received either vehicle alone or VAN administered orally once daily for five consecutive days. Animals were monitored daily for clinical signs of disease, including weight loss, behavioral changes, hunched posture, reduced activity, wet tail, and diarrhea. Fecal samples were collected on days 3 and 5 for *C. difficile* spore count, using pre-reduced BHIS agar supplemented with 0.2% taurocholic acid (32). To quantify the *C. difficile* toxin titer, fecal pellets were collected aseptically on day 3 and day 5 and suspended in sterile PBS, and the supernatants were collected for *C. difficile* toxin evaluation using the enzyme-linked immunosorbent assay (ELISA) assay kit (tgc BIOMICS) (63, 64). To assess the activity of the tested drugs in preventing *C. difficile* recurrence, treatments were discontinued after day 5, and mice were monitored for clinical relapse signs. Animals were humanely euthanized via CO₂ asphyxiation at the end of the experiment.

### Statistical analysis

The statistical analysis was conducted using GraphPad Prism version 10.0 for Windows (GraphPad Software, La Jolla, California).

## RESULTS

### Abafungin exhibited potent anti-*C*. *difficile* activity

**Fig 1:**
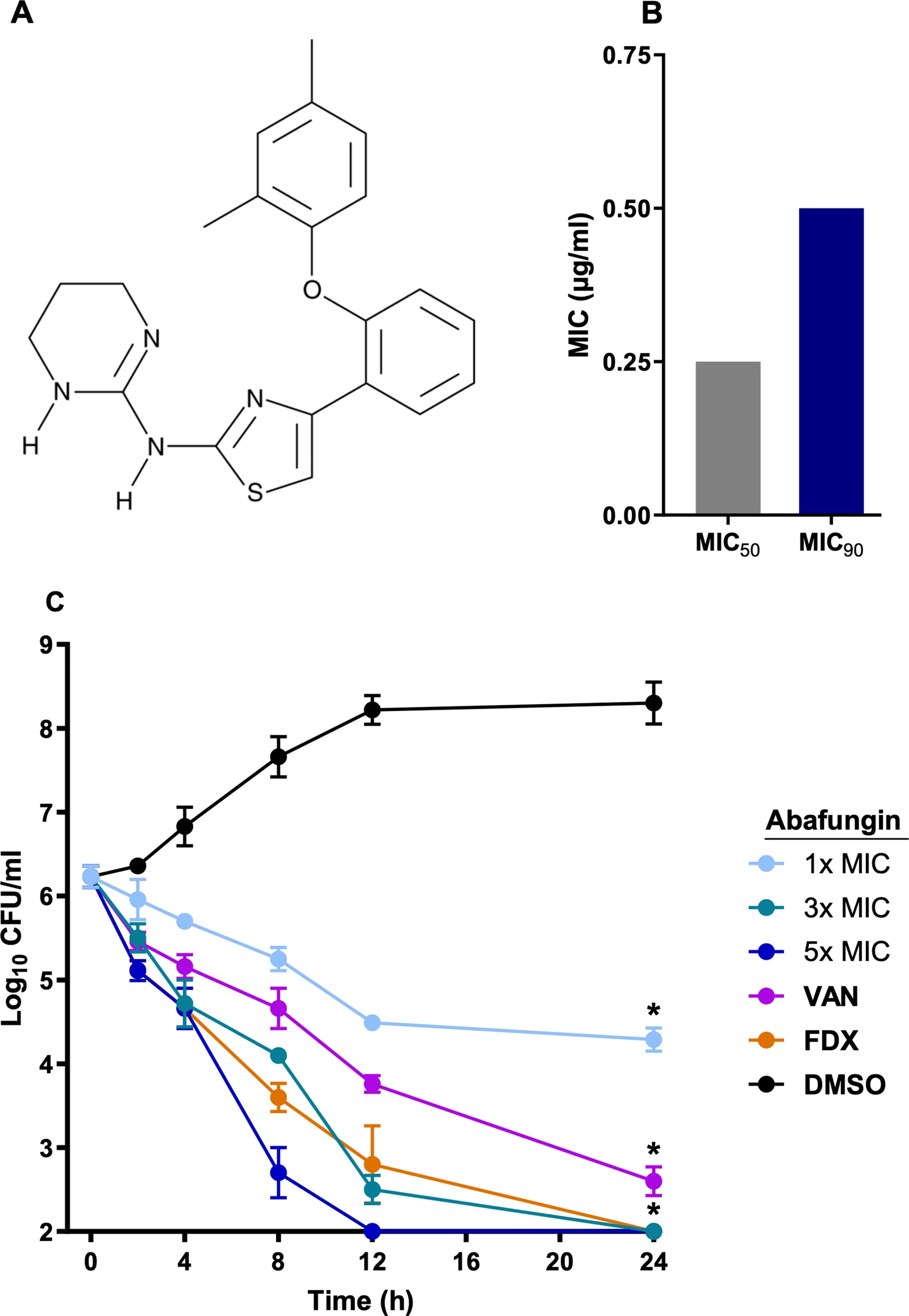
Anti-*C. difficile* activity of abafungin. (**A**) Chemical structure of abafungin. (**B**) Minimum inhibitory concentration (MIC_₅₀_ and MIC_₉₀_) values of abafungin determined against 32 clinical isolates of *C. difficile* tested in this study. (**C**) Killing kinetics activity of abafungin against *C. difficile* ATCC 43255 over 24 h in pre-reduced BHIS broth. Data represent the mean ± SD of three technical replicates from two independent biological experiments. Statistical significance was determined by two-way ANOVA with Dunnett’s multiple-comparisons test. Asterisks (*) denote significant differences relative to the DMSO control (*P* < 0.05).

In our previous drug repurposing study, screening of the Pharmakon 1600 and Johns Hopkins clinical compound libraries identified several azole antifungals with selective activity against *C. difficile* (65, 66). To further evaluate this class, we assessed the anti-*C*. *difficile* activity of a panel of antifungals (Tables S3). While most azoles exhibited limited or moderate activity (MICs ranging from 1 to >64 µg/mL), abafungin displayed the most potent activity with an MIC of ≤ 0.5 µg/mL, markedly lower than other agents tested. Based on its superior potency within the azole class, abafungin was selected for further investigation as a potential anti-*C*. *difficile* inhibitor, particularly given its reported sporicidal activity against fungal pathogens (26). To assess the anti-*C*. *difficile* activity of abafungin , we evaluated abafungin’s MICs against a panel of 32 clinical *C. difficile* isolates (Table 1). Abafungin demonstrated potent anti-*C*. *difficile* activity superior to that of the standard-of-care antibiotic, VAN. The MIC_₅₀_ and MIC_₉₀_ of abafungin were 0.25 μg/mL and 0.5 μg/mL, respectively (Fig. 1B). These values were lower than those of VAN (MIC_₅₀_ = 0.5 µg/mL; MIC_₉₀_ = 2 µg/mL) and were higher than those observed for FDX (MIC_₅₀_ = 0.015 µg/mL; MIC_₉₀_ = 0.06 µg/mL).

**TABLE 1:** MICs (μg/mL) of abafungin and reference antibiotics against *C. difficile* strains^*c*^

No.	*C. difficile* strains	Minimum inhibitory concentration (MIC) (μg/mL)		
		Abafungin	Control antibiotics	
			VAN^*a*^	FDX^*b*^
1	NR-49302	0.5	0.5	0.06
2	NR-49304	0.25	0.5	0.06
3	NR-49306	0.25	0.5	0.0078
4	NR-49307	0.25	0.5	0.0078
5	NR-49308	0.25	0.25	0.015
6	NR-49310	0.25	0.5	0.015
7	NR-49313	0.25	1	0.03
8	NR-49314	0.25	0.5	0.06
9	NR-49318	0.5	0.5	0.03
10	NR-49319	0.5	0.25	0.06
11	AR-1067	0.5	2	0.06
12	AR-1072	0.5	2	0.06
13	AR-1076	0.25	2	0.03
14	AR-1077	0.25	0.5	0.0078
15	AR-1078	0.25	1	0.03
16	AR-1079	0.25	0.5	0.0078
17	AR-1082	0.25	0.5	0.015
18	AR-1083	0.25	1	0.03
19	AR-1085	0.25	0.5	0.0078
20	AR-1086	0.5	0.25	0.0078
21	AR-1087	0.25	0.5	0.0078
22	AR-1088	0.25	0.5	0.015
23	AR-1089	0.25	0.25	0.06
24	AR-1090	0.25	0.5	0.03
25	AR-1092	0.5	2	0.015
26	AR-1093	0.25	0.25	0.015
27	AR-1094	0.25	0.25	0.03
28	AR-1095	0.5	2	0.03
29	AR-1096	0.25	0.25	0.015
30	ATCC 630	0.25	1	0.015
31	ATCC 43255	0.5	0.25	0.015
32	ATCC BAA-1870	0.5	1	0.0078

^*a*^ VAN, vancomycin.

^*b*^ FDX, fidaxomicin.

^*c*^ NR strains were obtained from BEI Resources (Biodefense and Emerging Infections Research Resources Repository). AR strains were obtained from the CDC (Centers for Disease Control and Prevention) and ATCC (American Type Culture Collection).

### Abafungin exhibited a rapid bactericidal activity against *C. difficile*

Abafungin’s killing kinetics were evaluated against a high inoculum of *C. difficile* using a 24-h time-kill assay at 1×, 3×, and 5× MIC. Abafungin exhibited concentration-dependent killing activity. At 1× MIC, abafungin showed a bacteriostatic effect, achieving an approximate 2-log₁₀ CFU/mL reduction after 24 h. At 3× MIC, abafungin induced a >3-log₁₀ reduction in viable bacterial counts within 12 h and resulted in complete bacterial clearance by 24 h, demonstrating activity comparable to VAN and FDX. At 5× MIC, abafungin rapidly eradicated *C. difficile*, achieving complete clearance within 12 h and surpassing the killing kinetics of both VAN and FDX (Fig. 1C).

### Abafungin showed limited activity against representative isolates of gut microbiota

As depicted in Table 2, abafungin demonstrated limited activity against the tested isolates of the common gut bacteria such as *Bacteroides*, *Bifidobacterium*, *Lactobacillus*, and *Veillonella* strains with MIC values ranging from 16 to >256 (64-fold to >1,024-fold its MIC_50_ against *C. difficile*) (Table 2). In contrast, VAN and FDX exhibited potent inhibition of *Bifidobacterium* strains with MIC values ranging from ≤2 to 8 μg/mL. Further, FDX exhibited activity against *Lactobacillus* strains at a MIC range of ≤2 to 8 μg/mL (except for *Levilactobacillus brevis* which has an MIC of 64 μg/mL), whereas VAN showed negligible inhibition (MIC ≥256 μg/mL) against most *Lactobacillus* isolates. An exception was *L. gasseri*, which remained susceptible to VAN, exhibiting an MIC of ≤2 µg/mL. On the other hand, VAN and FDX had an MIC range of 32 to 256 µg/mL against *Bacteroides* strains. Taken together, these findings demonstrate that abafungin possesses a selectivity profile comparable to current standard-of-care therapies while maintaining limited activity against representative members of the commensal gut microbiota tested in the study.

**TABLE 2:** MICs of abafungin (μg/mL) and reference antibiotics against representative isolates of gut microbiota

No.	Representative isolates of gut microbiota	Minimum inhibitory concentration (MIC) (μg/mL)		
		Abafungin	VAN	FDX
1	*Bifidobacterium longum* HM-846	32	8	≤2
2	*Bifidobacterium angulatum* HM-1189	32	≤2	≤2
3	*Bifidobacterium longum* HM-847	16	≤2	≤2
4	*Bifidobacterium breve* ATCC 15700	32	≤2	≤2
5	*Bifidobacterium longum* HM-845	32	≤2	≤2
6	*Bifidobacterium longum* HM-848	16	8	≤2
7	*Bacteroides finegoldii* HM-727	16	64	>256
8	*Bacteroides stercoris* HM-1036	64	128	>256
9	*Bacteroides fragilis* HM-711	64	256	>256
10	*Bacteroides fragilis* HM-709	32	256	>256
11	*Bacteroides fragilis* HM-714	16	32	>256
12	*Lacticaseibacillus rhamnosus* ATCC 53103	256	>256	4
13	*Lacticaseibacillus rhamnosus* HM-106	32	>256	2
14	*Lactobacillus gasseri* ATCC 19992	64	≤2	≤2
15	*Lactobacilius casei* ATCC 334	128	>256	4
16	*Levilactobacillus brevis* ATCC 14869	16	>256	64
17	*Lactobacillus* sp. HM-100	>256	>256	8
18	*Veillonella* sp. HM-49	>256	>256	32

### Abafungin is not cytotoxic to mammalian cells

**Fig 2:**
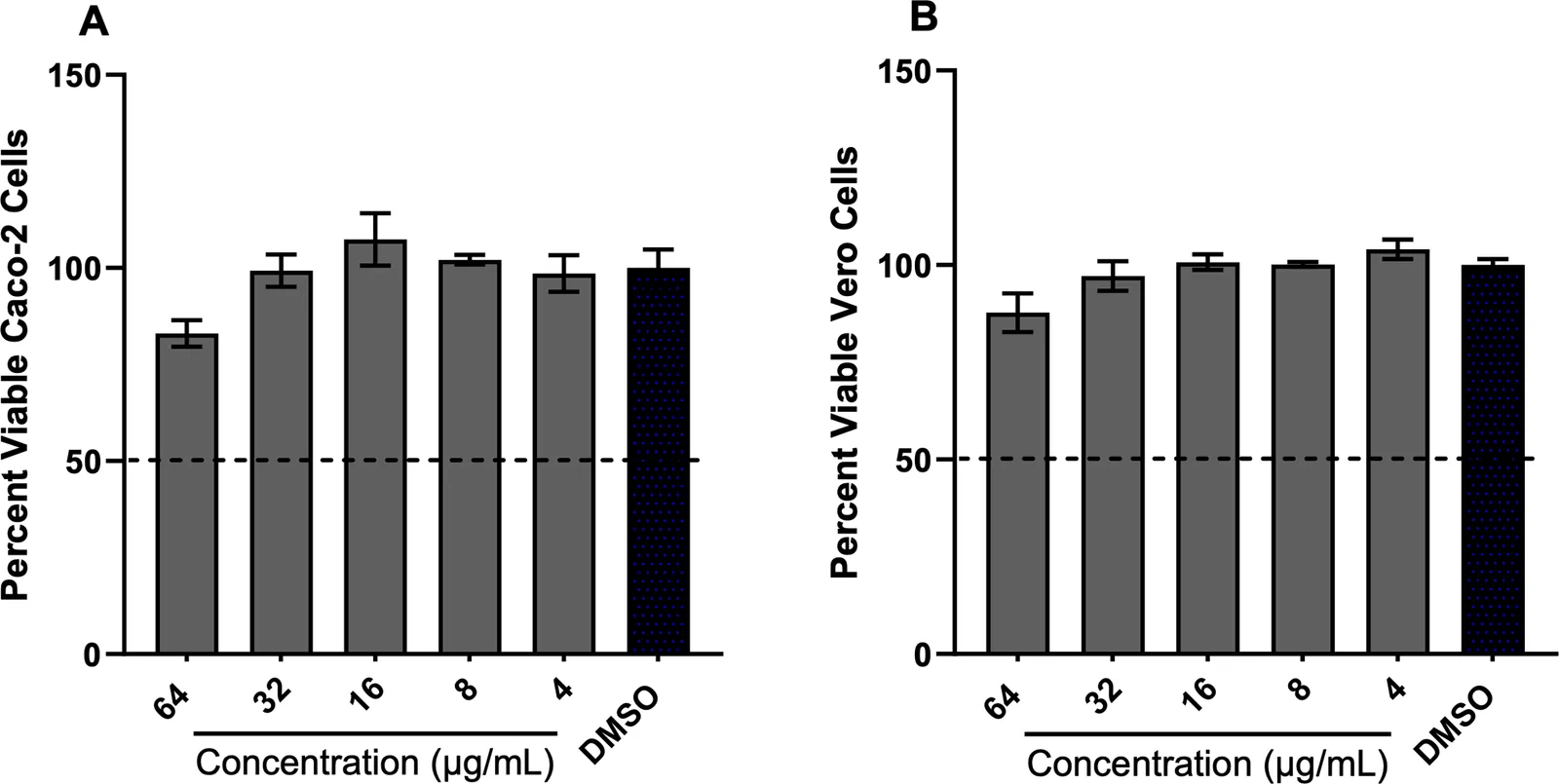
Abafungin’s cytotoxicity against mammalian cell lines. The MTS (3-(4,5-dimethylthiazol-2-yl)-5-(3-carboxymethoxyphenyl)-2-(4-sulfophenyl)-2H-tetrazolium) assay was used to examine the cytotoxicity of abafungin beginning at concentrations ranging from 4 to 64 μg/mL. Cell viability was determined relative to the DMSO-treated control in (**A**) Caco-2 cells and (**B**) Vero cells. Data are presented as mean ± SD from three technical replicates performed in two independent biological experiments.

To assess the therapeutic safety window of abafungin, cytotoxicity studies were conducted using human colorectal epithelial cells (Caco-2) (Fig. 2A) and African green monkey kidney cells (Vero) (Fig. 2B). Following 24 h of drug exposure, cell viability remained comparable to the DMSO control at concentrations up to 64 µg/mL (256 × its MIC_50_ against *C. difficile*) (Fig. 2).

### Abafungin suppresses *C. difficile* toxin production

**Fig 3:**
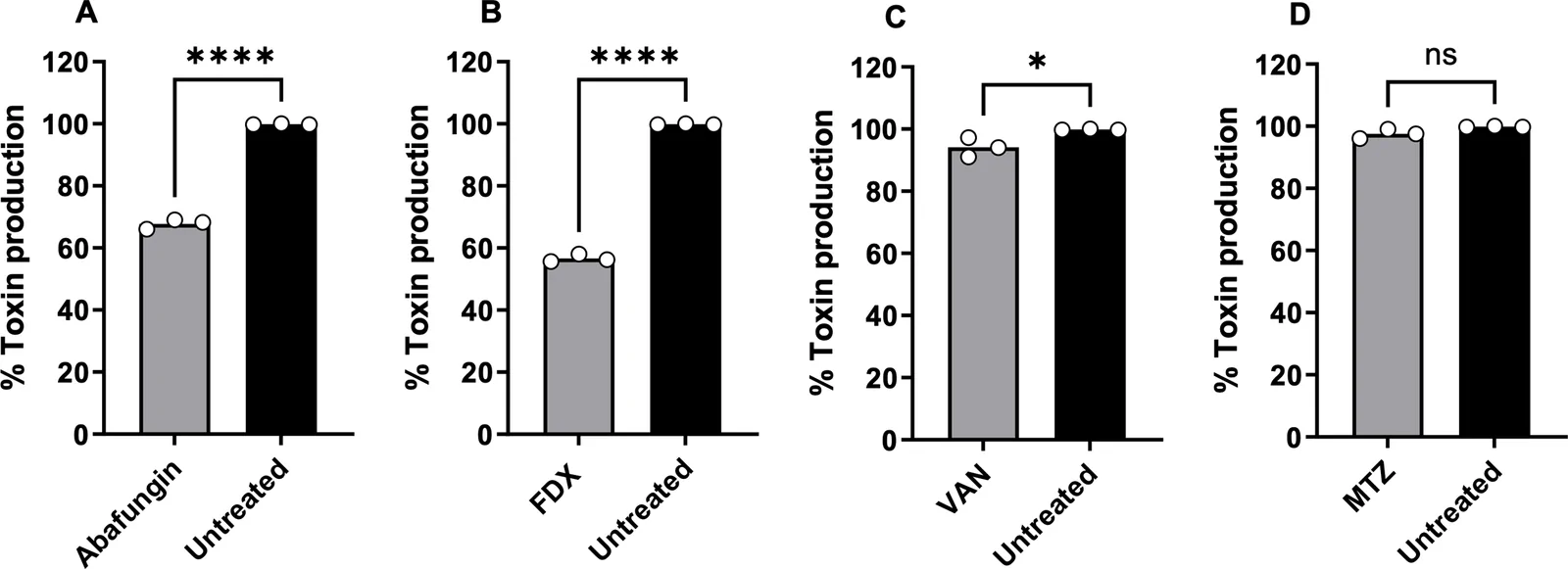
Toxin inhibition activity of abafungin**.** *C. difficile* ATCC 43255 cells were treated with either; Abafungin (**A**), FDX (**B**), VAN (**C**), and MTZ (**D**) at 0.25× MIC. ELISA was used to determine the toxin level in each supernatant (OD_450_ − OD_620_). Data are presented as percentage of toxin production (mean ± SD) from three technical replicates performed in two independent biological experiments. The data were analyzed via one-way ANOVA with post hoc Dunnett’s test for multiple comparisons. Asterisks (*) denote statistically significant differences for treatment with test agents compared with the negative control; * (*P* < 0.05), **** (*P* < 0.0001), ns, not significant.

To determine whether abafungin could suppress *C. difficile* virulence, we evaluated its ability to inhibit toxin production at sub-inhibitory concentrations. Cultures of *C. difficile* ATCC 43255 were exposed to 0.25× MIC of abafungin, FDX, VAN, or MTZ, and culture supernatants were analyzed by ELISA for TcdA and TcdB levels. Importantly, treatment with any of the tested agents at 0.25× MIC did not affect bacterial counts relative to the DMSO control (Fig. S1), ensuring that changes in toxin levels were not attributable to reduced bacterial count. Abafungin significantly impaired toxin production, reducing combined TcdA/TcdB levels by approximately 31.8%. This inhibitory activity was comparable to that of FDX, which produced ~43.8% reduction (Fig. 3). In contrast, VAN and MTZ showed toxin levels comparable to that of untreated control.

### Abafungin inhibits *C. difficile* sporulation and germination

**Fig 4:**
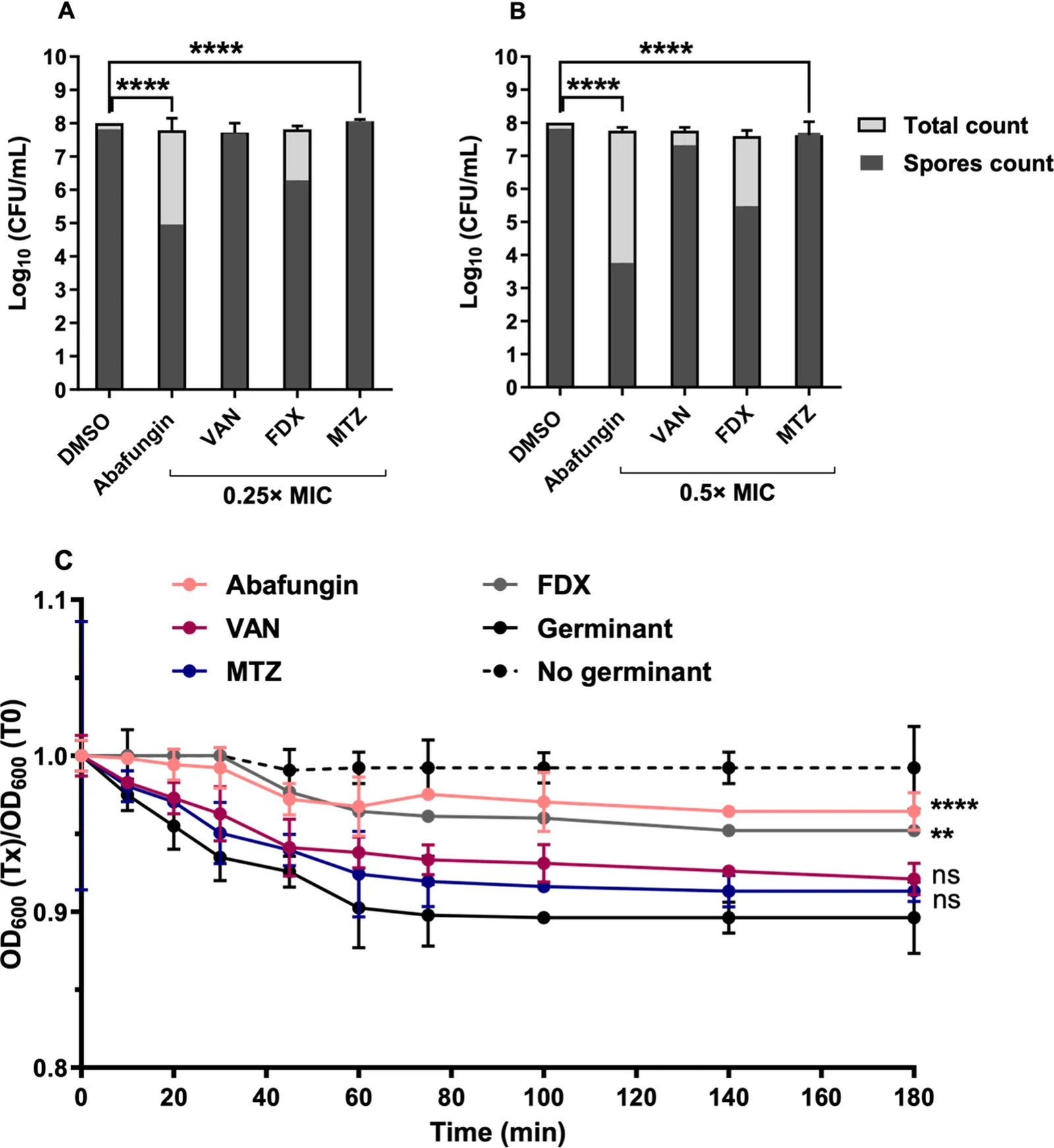
Inhibition of *C. difficile* sporulation, germination, and spore outgrowth by abafungin. (**A and B**) Sporulation inhibition. *C. difficile* was incubated with abafungin, FDX, VAN, or MTZ at 0.25× MIC (**A**) or 0.5× MIC (**B**) for 6 days. DMSO served as a vehicle control. Following incubation, aliquots were taken, diluted, and plated to determine the total counts. Then, cultures were heated at 65°C for 40 min, serially diluted, and plated onto pre-reduced BHIS agar supplemented with 0.1% taurocholic acid for enumeration of spores (log₁₀ CFU/mL). The data are presented as average (triplicate samples for each treatment) ± standard deviation. The data of the spore count were analyzed via two-way ANOVA with post hoc Dunnett’s test for multiple comparisons (*P* < 0.0001). (**C**) Spore germination inhibition. Purified spores were exposed to abafungin, FDX, VAN, or MTZ in the presence of germinants, and germination was monitored by loss of optical density at 600 nm. The ratio of the OD_600_ at different time points (*T*_*x*_) to the OD_600_ at time zero (*T*_0_) was plotted against time. Data are presented as mean ± SD from three technical replicates performed in two independent biological experiments. The data were analyzed via a two-way ANOVA with post hoc Dunnett’s test for multiple comparisons. An asterisk (*) indicates a statistically significant difference between treatment with test agents and the germinant control; ** (*P <* 0.0011), **** (*P* < 0.0001). The experiment was performed in duplicate (three technical replicates for each).

We next evaluated the impact of abafungin on *C. difficile* spore formation, germination, and outgrowth. Exposure of *C. difficile* to sub-inhibitory concentrations of abafungin (0.25× MIC) for 6 days resulted in a significant reduction in spore burden, producing an approximate 2-log₁₀ decrease in spore counts, demonstrating activity comparable to that of FDX. Notably, the total counts remained unchanged relative to the DMSO-treated negative control, indicating a selective effect on sporulation rather than bacterial growth (Fig. 4A). At 0.5× MIC, abafungin reduced spore counts by approximately 4-log₁₀. This activity was superior to that observed with FDX, which achieved an approximately 2.3-log₁₀ reduction (Fig. 4B). VAN and MTZ, in contrast, were incapable of interfering with *C. difficile* sporulation when used at the same concentrations.

We next evaluated the ability of abafungin to interfere with *C. difficile* spore germination, a critical phase in the pathogen’s life cycle during which dormant spores transition into toxin-producing vegetative cells. Spores of *C. difficile* ATCC 43255 were exposed to abafungin in the presence of germinants (0.2% taurocholic acid and 50 mM glycine). At 5× MIC, abafungin substantially reduced the germination rate, as reflected by a minimal decrease in optical density over time (OD₆₀₀ ~0.97), indicating pronounced inhibition of the transition from spores to vegetative cells, comparable to the no-germinant control (Fig. 4C). FDX exhibited partial inhibitory activity at 5× MIC (OD₆₀₀ ~0.95), in agreement with its known anti-germinant properties. However, VAN and MTZ showed no significant suppression of germination, with OD₆₀₀ values similar to germinant-only controls.

### Abafungin disrupts metal ion homeostasis in *C. difficile* and interferes with metabolic pathways that require metal cofactors

**Fig 5:**
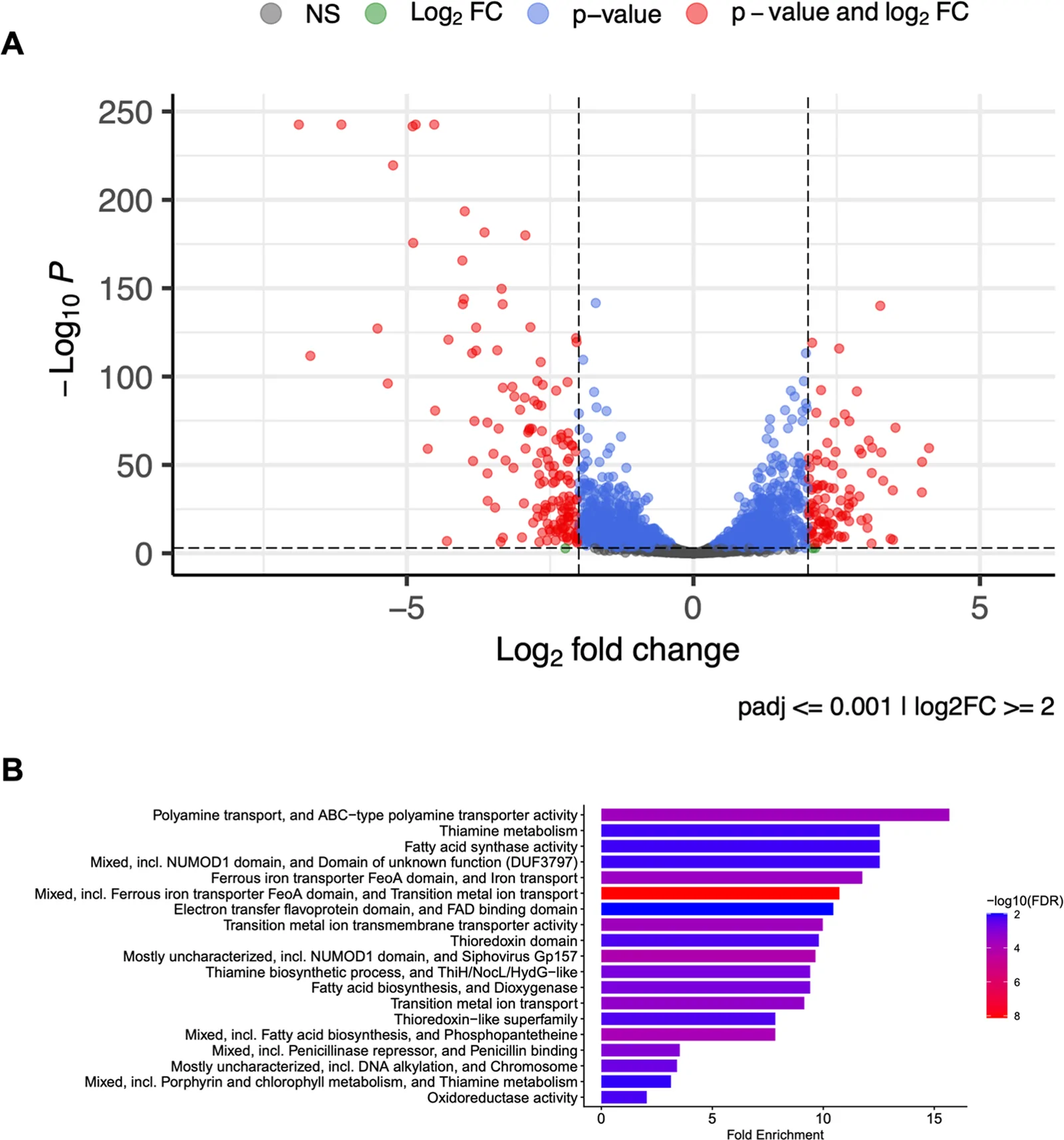
RNA sequencing analysis of *C. difficile* following treatment with abafungin. (**A**) Volcano plot illustrating global differential gene expression in *C. difficile* after treatment with abafungin compared with untreated control. Each dot represents a gene, plotted according to log₂ fold change and −log₁₀ adjusted *P*-value (padj). Genes meeting the significance threshold (adjusted *P*-value (padj) ≤ 0.001 and |log₂ fold change| ≥ 2) are highlighted in red. (**B**) Gene ontology (GO) enrichment analysis of significantly differentially expressed genes. The bar chart displays the top enriched functional categories ranked by fold enrichment, with color intensity corresponding to statistical significance (−log₁₀ FDR). Pathways related to metal ion transport and metal homeostasis were among the most significantly enriched processes. RNA-seq analysis was performed using three independent biological replicates per condition.

Transcriptomic profiling of *C. difficile* following treatment with abafungin revealed extensive global transcriptional remodeling, as illustrated by the volcano plot (Fig. 5A). Using a threshold of log₂ fold change ≥ 2 and adjusted *P*-value (padj) ≤ 0.001, numerous genes were significantly upregulated or downregulated relative to untreated controls, indicating a strong cellular response to abafungin exposure. Functional enrichment analysis highlighted metal ion transport and metal homeostasis as the most significantly affected biological processes, exhibiting the highest enrichment scores and strongest statistical significance (−log₁₀ FDR) among all pathways identified. Specifically, gene ontology (GO) categories related to transition metal ion transport (Fig. 5B), including metal ion binding (e.g., CD630_RS17575, CD630_RS16150, CD630_RS19170, CD630_RS01395, CD630_RS14340, CD630_RS06465, CD630_RS08355, CD630_RS09175, CD630_RS08345, CD630_RS08085, CD630_RS06610, CD630_RS13115, CD630_RS11295, CD630_RS09285, CD630_RS09265), FeoA-associated transport systems (e.g., CD630_RS01400, CD630_RS08985, CD630_RS08100, and CD630_RS08105), magnesium ion binding (e.g., CD630_RS18060 and CD630_RS09290), transmembrane metal transporters (e.g., CD630_RS02175, CD630_RS11470, CD630_RS09270, CD630_RS02180, CD630_RS08115, CD630_RS08110, CD630_RS08980, CD630_RS06530, CD630_RS09505, and CD630_RS08975), and metallopeptidase (e.g., CD630_RS04020, CD630_RS12645, CD630_RS15150, and CD630_RS04050), exhibited significant transcriptional changes (Fig. 5B). Disruption of these pathways likely affects essential metal cofactors required for key enzymatic systems, including oxidoreductases and flavoprotein-associated electron transfer processes (67–69), and these pathways were also enriched in the GO analysis. These findings suggest that abafungin may influence pathways involved in metal acquisition and intracellular metal homeostasis which play critical roles in bacterial metabolism, enzymatic activity, and redox homeostasis.

### Magnesium supplementation reduces the susceptibility of *C. difficile* to abafungin

**Fig 6:**
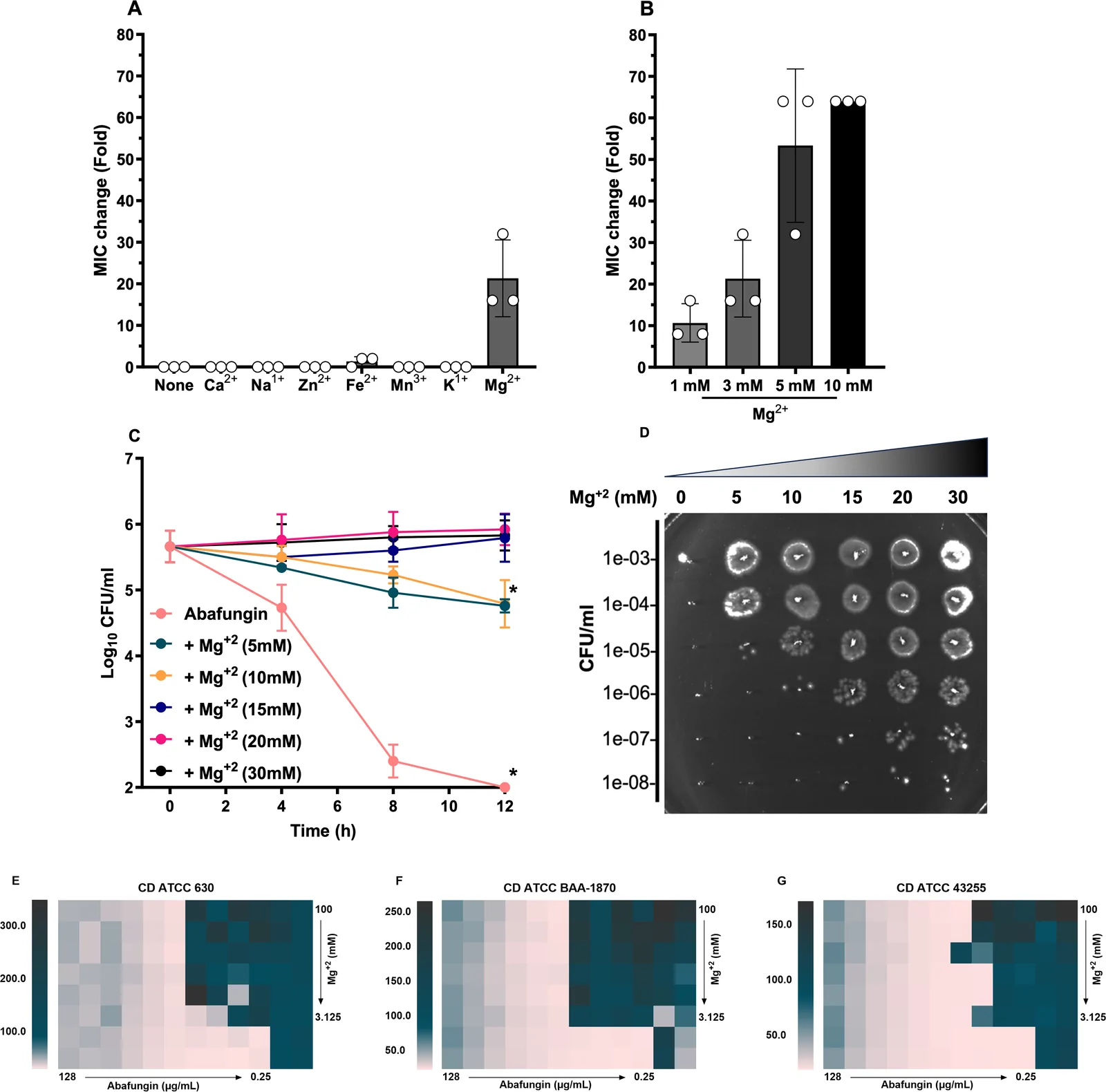
Magnesium supplementation antagonizes the anti-*C. difficile* activity of abafungin. (**A**) MIC determination in the presence of individual cations. The MIC of abafungin against *C. difficile* ATCC 43255 was determined in minimal *C. difficile* medium supplemented with individual cations (Ca²^+^, Na^+^, Zn²^+^, Fe²^+^, Mn²^+^, K^+^, or Mg²^+^; 3 mM each). (**B**) MIC determination of abafungin in minimal medium supplemented with increasing concentrations of Mg²^+^. (**C**) Time-kill kinetics of abafungin in the presence of Mg²^+^. *C. difficile* cultures were exposed to abafungin at 5× MIC in the absence or presence of MgCl₂ (5–30 mM). Statistical significance was determined by two-way ANOVA with Dunnett’s multiple-comparisons test. Asterisks (*) denote significant differences relative to the DMSO control (*P* < 0.05). (**D**) Spot-plating assay corresponding to the time-kill experiment showing bacterial survival under the same conditions. (**E–G**) Checkerboard assay evaluating the interaction between abafungin and Mg²^+^. FICI analysis demonstrated a pronounced antagonistic interaction, with Mg²^+^ against *C. difficile* ATCC 630 (**E**), *C. difficile* ATCC BAA-1870 (**F**), and *C. difficile* ATCC 43255 (**G**). Data are presented as mean ± SD from three technical replicates performed in two independent biological experiments.

Given that transcriptomic analysis indicated significant perturbation of genes involved in metal ion transport and metal homeostasis in *C. difficile* following treatment with abafungin, we next examined whether supplementation with exogenous metal cations could influence the antibacterial activity of abafungin. To this end, *C. difficile* cultures grown in CDMM were supplemented with different cations (Ca²^+^, Na^+^, Zn²^+^, Fe²^+^, Mn²^+^, K^+^, Mg²^+^ [3 mM]), and the MIC of abafungin was determined in the presence and absence of these cations. Supplementation with Mg²^+^ resulted in a marked increase in the MIC of abafungin, producing a 16-fold reduction in anti-*C. difficile* activity, whereas the other tested cations had no significant effect (Fig. 6A). We therefore further investigated the effect of increasing Mg²^+^ concentrations (1, 3, 5, and 10 mM) on the activity of abafungin. Abafungin’s MICs increased in a dose-dependent manner, with supplementation of 1 and 3 mM Mg²^+^ resulting in 8- and 16-fold increases in the MIC of abafungin, respectively. Higher Mg²^+^ concentrations (5 and 10 mM) produced an even greater effect, leading to a 64-fold increase in the MIC (Fig. 6B). These findings were corroborated in time-kill assays. Treatment with abafungin (at 5× MIC) alone achieved complete eradication of *C. difficile* within 12 h, whereas supplemental Mg²^+^ progressively attenuated the bactericidal activity of abafungin. In the presence of 5–10 mM MgCl₂, abafungin’s bactericidal effect was reduced to <1-log₁₀ CFU/mL decline, whereas at higher Mg²^+^ concentrations (15, 20, and 30 mM), bacterial count remained unchanged over the course of the experiment (Fig. 6C and D). To further confirm the Mg²^+^-dependent antagonism, a checkerboard assay was performed. Consistent with the MIC and time-kill findings, Mg²^+^ at 3.1 mM increased the MIC of abafungin from 0.25 to 2–4 µg/mL against the three tested strains, corresponding to an 8- to 16-fold reduction in potency (Fig. 6E through G). Collectively, these results demonstrate a robust antagonistic effect of Mg²^+^ on abafungin activity, characterized by a clear dose-dependent relationship.

### Abafungin interacts synergistically with VAN and MTZ

**Fig 7:**
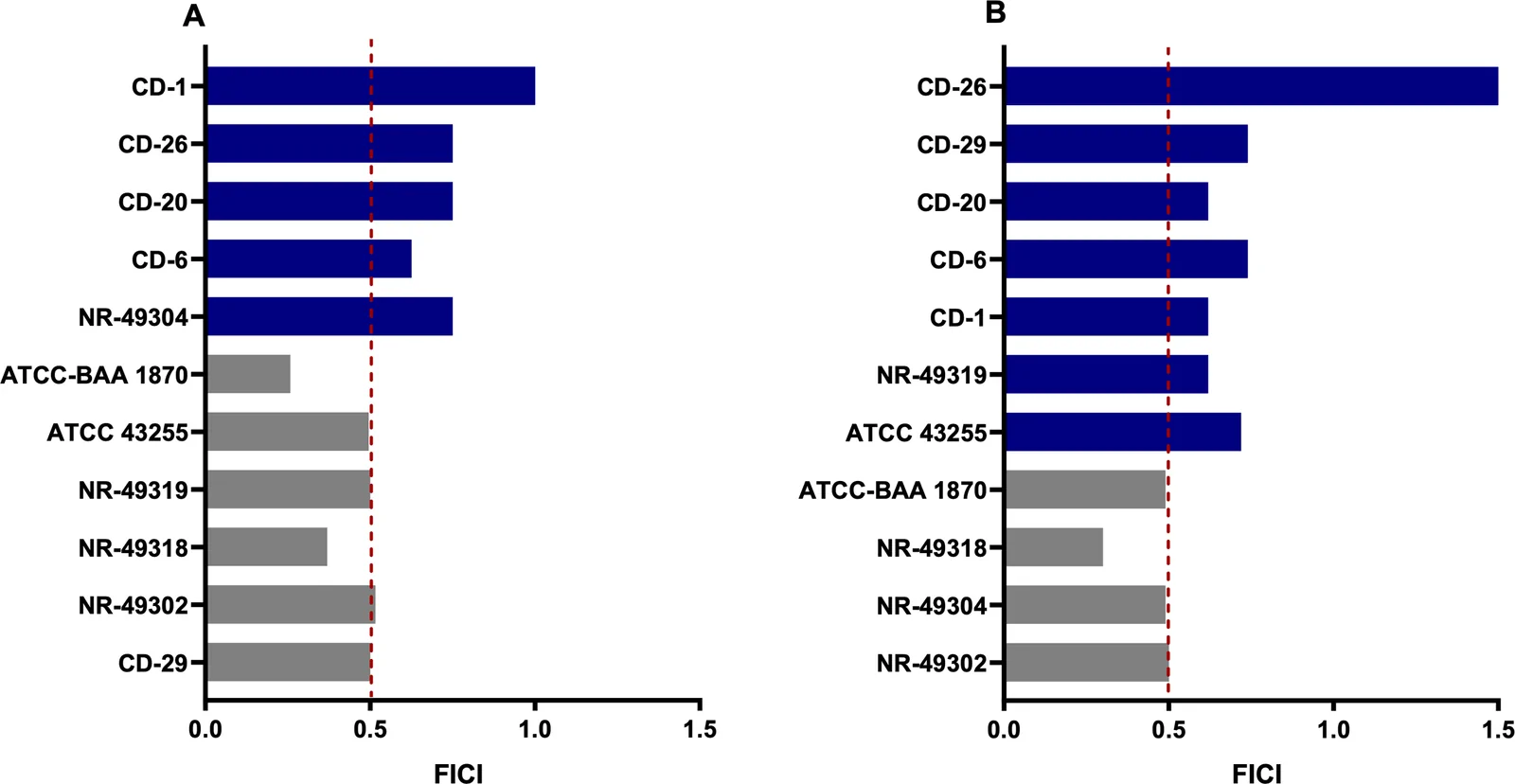
Checkerboard assay of abafungin with control antibiotics. FICI demonstrating the interactions of abafungin with VAN (**A**) and MTZ (**B**) against *C. difficile* strains. The gray bars show the synergistic interactions of abafungin.

We next investigated whether abafungin could potentiate the efficacy of VAN and MTZ. Checkerboard assays were performed against 11 clinically relevant *C. difficile* isolates, and the FICI was calculated to define drug–drug interactions. As shown in Fig. 7A, abafungin exhibited a robust synergistic interaction with VAN, achieving FICI values ≤ 0.5 in six out of 11 isolates tested. A synergistic enhancement was also observed with MTZ in four out of the 11 isolates (Fig. 7B).

### The activity of abafungin is not affected by simulated gastrointestinal conditions

Considering abafungin’s potential use as an orally administered CDI therapeutic, its functional stability within the harsh physiological environment of the gastrointestinal tract was examined. The anti-*C. difficile* activity of abafungin was evaluated after exposure to SGF and SIF, conditions that mimic the pH and enzymatic composition of the digestive system. MIC values against two clinical *C. difficile* isolates were measured at 0, 4, and 24 h of incubation. As shown in Table 3A, abafungin retained full anti-*C. difficile* activity following 24 h exposure to SGF, with no significant increase in MIC values. VAN and FDX displayed unchanged or only one-fold shifts under the same conditions, indicating comparable stability. Likewise, exposure to SIF had no detrimental effect on the anti-*C. difficile* activity of abafungin comparing to VAN amd FDX (Table 3B).

**TABLE 3:** MICs of abafungin (μg/mL) and reference antibiotics against *C. difficile* in the presence of simulated gastrointestinal fluids (SGF/ SIF)

Simulated gastric fluid (SGF)									
*C. difficile* strains	Abafungin			VAN			FDX		
	0 h	4 h	24 h	0 h	4 h	24 h	0 h	4 h	24 h
ATCC 43255	0.5	0.5	1	0.25	0.5	0.5	0.015	0.03	0.03
ATCC BAA-1870	0.5	0.5	0.5	1	1	1	≤0.0078	0.015	0.03
									
**Simulated intestinal fluid (SIF)**									
*C. difficile* strains	Abafungin			VAN			FDX		
	0 h	4 h	24 h	0 h	4 h	24 h	0 h	4 h	24 h
ATCC 43255	0.5	0.5	0.5	0.25	0.5	1	0.015	0.015	0.03
ATCC BAA-1870	0.5	0.5	0.5	1	1	2	≤0.0078	≤0.0078	0.015

### Abafungin in combination with VAN reduces CDI recurrence *in vivo*

**Fig 8:**
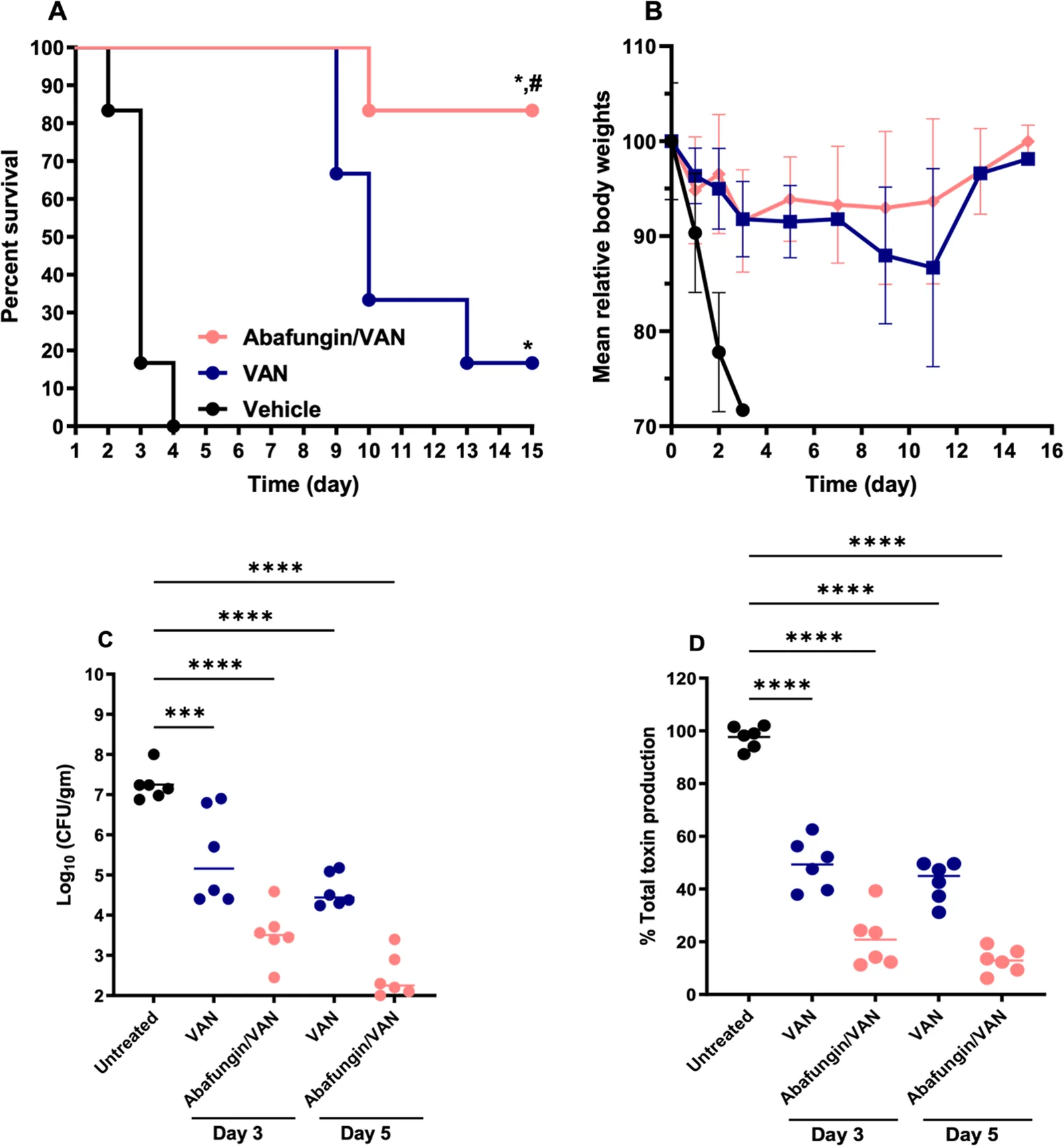
Efficacy of abafungin in combination with VAN in a mouse model of recurrent CDI. Oral dosages of abafungin (20 mg/kg) and VAN (10 mg/kg) in combination, VAN (10 mg/kg) alone, or vehicle were administered to mice (*n* = 6/group) once daily for 5 days beginning 2 h after infection. (**A**) Kaplan–Meier survival plots after treatment with abafungin and controls. Log-rank test was used to compare the survival distributions between the study groups. (*) Denotes significant difference from the vehicle group (*P* < 0.05) and (#) denotes significant difference from the VAN-treated group (*P* < 0.05). (**B**) Body weights during the study; the VAN group showed recurrence of infection on days 10 to 13. (**C**) Spore counts in fecal samples collected on days 3 and 5 of the experiment. Treatment with abafungin/VAN combination resulted in a significant reduction of spore counts (>5-log_10_ reduction). (**D**) The levels of *C. difficile* toxins in mice fecal pellets collected on days 3 and 5 of the experiment measured using an ELISA kit. Data are presented as mean ± SD. Statistical analysis was performed using one-way ANOVA with appropriate post hoc testing; *** (*P* < 0.0007) and **** (*P* < 0.0001).

Given the potent anti-virulence activity of abafungin observed *in vitro* against *C. difficile*, we next evaluated the effect of abafungin administration in a *C. difficile* recurrent infection mouse model in the context of vancomycin treatment. After infection, mice were randomized to receive either VAN alone (10 mg/kg, PO) or co-administration of VAN (10 mg/kg) with abafungin (20 mg/kg, PO) once daily for five consecutive days. Consistent with its clinical limitations, VAN monotherapy resulted in suboptimal protection, with only 16.6% (1 out of 6 mice) surviving from CDI recurrence within the 15-day observation period. In striking contrast, the abafungin/VAN combination yielded markedly improved outcomes, achieving 83.3% survival (five out of six mice) (Fig. 8A). Analysis of body weight trajectories provided a clear indication of treatment efficacy during acute *C. difficile* infection (Fig. 8B). Vehicle-treated mice exhibited progressive weight loss beginning on day 2 and succumbed to infection by day 3, reflecting the fulminant course of untreated CDI. In contrast, mice receiving VAN did not show a decrease in body weights initially, but subsequently displayed weight loss coincident with disease relapse between days 8 and 10. Only one out of six animals (16.6%) survived long term, with body weight gradually returning to baseline after day 11. Mice treated with the combination of abafungin and VAN showed an early decline in weight similar to the VAN group through day 3. However, all animals thereafter demonstrated steady and continuous weight recovery for the remainder of the study. These findings indicate that adjunctive abafungin therapy improves overall clinical outcome and reduces recurrence severity relative to VAN monotherapy.

Fecal spore quantification demonstrated the mechanistic basis for this protection. By day 3, animals receiving the combination therapy exhibited a pronounced reduction in spore burden (3.5 × 10⁴ CFU/g), compared with 1 × 10⁸ CFU/g in vehicle-treated mice and 1 × 10⁶ CFU/g in the VAN group (Fig. 8C). Spores were reduced in the combination-treated group to ~2.4 × 10^2^ CFU/g), whereas recurrent shedding was observed in VAN-treated animals following drug withdrawal (~6 × 10⁵ CFU/g). Fecal toxin quantification further supported the enhanced therapeutic activity of the combination regimen. Toxin levels in fecal pellets collected on days 3 and 5 revealed that mice receiving abafungin plus VAN had markedly reduced toxin burden compared with both untreated controls and VAN-treated mice (Fig. 8D). VAN monotherapy was associated with substantial toxin levels, approximately 50% on day 3 and 40% on day 5. In contrast, the combination treatment lowered fecal toxin titers to ~20% and ~15% on days 3 and 5, respectively. These data indicate that abafungin, in the context of combination treatment with VAN, contributes to reduced toxin production and decreased spore persistence *in vivo*, which may collectively enhance overall disease control.

## DISCUSSION

CDI remains a major public health burden in the United States and worldwide, particularly among hospitalized patients receiving broad-spectrum antimicrobial therapy (70). This highlights the need for novel therapeutic strategies capable of targeting multiple stages of the pathogen’s life cycle, including vegetative growth, toxin production, and spore formation. In this study, we investigated the anti-*C. difficile* potential of abafungin, an antifungal drug previously reported to exhibit potent sporicidal activity against fungal pathogens (26–28). Given the critical role of spores in CDI transmission, persistence, and recurrence, these properties provided a compelling rationale for exploring abafungin as a potential anti-*C. difficile* therapeutic lead. To our knowledge, this study is the first to demonstrate the antibacterial activity of abafungin against *C. difficile*.

Abafungin inhibited 90% of *C. difficile* isolates (MIC_90_) at the concentration of 0.5 μg/mL, which was comparable to current standards of care therapies. Interestingly, abafungin maintained strong activity against clinically significant hypervirulent ribotypes, including NAP1/BI/027 and ribotype 012 strains, which are frequently associated with severe disease outcomes and reduced susceptibility to conventional antibiotics. Additionally, abafungin demonstrated minimal inhibitory activity against representative commensal gut bacteria, suggesting a reduced potential to disrupt the intestinal microbiota, an important consideration given the strong association between microbiome dysbiosis and CDI recurrence. Furthermore, evaluation of killing kinetics under high-inoculum conditions demonstrated that abafungin exerts concentration-dependent bactericidal activity, resulting in substantial reductions in viable bacterial counts over time. These findings collectively highlight the potent anti-*C. difficile* activity of abafungin and support its potential utility as a useful candidate for the treatment of CDI.

Having established the potent anti-*C. difficile* activity of abafungin, we next investigated its ability to interfere with the two major drivers of *C. difficile* pathogenesis and recurrence: toxin production and spore formation. TcdA and TcdB are responsible for the hallmark intestinal epithelial injury in CDI, while spores, in contrast, mediate long-term persistence and transmission within the host and environment and are considered the primary cause of CDI relapse following standard antibiotic therapy. Thus, therapeutic agents that simultaneously suppress toxin synthesis and disrupt spore formation have the potential to greatly improve patient outcomes (71, 72). At sub-inhibitory concentrations, abafungin significantly reduced TcdA/TcdB levels by approximately 31.8%, an effect comparable to FDX, the known drug of choice for its ability to inhibit toxin production (73, 74). In parallel, abafungin markedly reduced spore formation by ~4-log_10_ CFU/mL, indicating strong antivirulence activity that extends beyond growth suppression. Because spore germination is the critical step for both CDI onset and recurrence, triggered by host-derived bile acids and co-germinants, agents capable of blocking spore transition into vegetative cells may interrupt the pathogenic cycle altogether (75, 76). In addition to its antibacterial activity, abafungin inhibited *C. difficile* spore germination and was compared with clinically used antibiotics in this regard. This complementary activity is particularly relevant to *C. difficile* pathogenesis, as standard therapies such as VAN and MTZ have limited ability to suppress toxin production or interfere with sporulation. By combining potent antimicrobial activity with attenuation of key virulence processes, abafungin may represent a promising candidate for combination therapeutic strategies aimed at improving treatment outcomes and reducing disease recurrence. Although the combination of VAN and MTZ has occasionally been used in severe CDI, clinical studies have not demonstrated improved outcomes compared with monotherapy (77). However, recent evidence suggests that pairing VAN with agents possessing complementary mechanisms, such as nifuroxazide, may enhance therapeutic efficacy and accelerate recovery (78). Consistent with this concept, checkerboard assays in the present study revealed synergistic interactions between abafungin and VAN, as well as between abafungin and MTZ. Such complementary activity may help compensate for the minimal limitations of current standard-of-care antibiotics, particularly their limited capacity to suppress toxin production and spore formation, which are two major drivers of CDI persistence and relapse.

To further explore the molecular basis underlying the anti-*C. difficile* activity of abafungin, transcriptomic analysis was performed in *C. difficile* following drug exposure. RNA-seq profiling revealed significant alterations in the expression of genes involved in metal ion transport and metal-dependent metabolic pathways. In particular, pathways associated with transition metal transport, iron acquisition systems, and metal-dependent enzymatic processes were among the most significantly affected. Given the essential role of metal ions as essential cofactors for numerous bacterial enzymes, including those involved in central metabolism, oxidative stress defense, and DNA synthesis, disruption of these pathways may impair critical cellular processes and contribute to the antibacterial activity of abafungin. These findings suggest that perturbation of metal ion homeostasis may contribute to the anti-*C. difficile* activity of abafungin and prompted further investigation of the impact of exogenous metal ions on its antibacterial activity.

Supplementation with different metal ions in a rescue assay revealed magnesium as a potent and selective antagonist of abafungin’s activity against *C. difficile*. The steep, concentration-dependent increase in MIC values, coupled with the near-complete loss of bactericidal activity at physiologically relevant Mg²^+^ concentrations, strongly suggests that Mg²^+^ modulates abafungin’s interaction with the bacterial cell. Such antagonism may arise from Mg²^+^-mediated stabilization of the cell envelope, alterations in drug uptake or membrane partitioning, or direct competition with abafungin at its molecular target (79–81). Collectively, these results support a model in which abafungin exerts anti-*C. difficile* effects, at least in part, by disrupting metal ion homeostasis (particularly magnesium) and impairing metal-dependent metabolic functions.

Prior to *in vivo* evaluation, the developability of abafungin was further assessed by examining its stability under simulated gastrointestinal conditions. Exposure to SGF and SIF did not alter the MIC values, indicating that abafungin retains antibacterial activity under physiologically relevant gastrointestinal environments, an important consideration for orally administered CDI therapies. In addition, cytotoxicity studies indicated a favorable safety profile, with >256-fold selectivity relative to the MIC_50_ value, underscoring a favorable selectivity profile and good safety margin for gastrointestinal therapeutic application.

Consistent with its antivirulence activity observed *in vitro*, abafungin in combination with VAN improved CDI outcomes in a murine model of recurrent *C. difficile* infection. Recurrence following treatment with VAN remains a major clinical limitation, largely due to the persistence of spores and continued toxin-mediated intestinal damage after antibiotic withdrawal. In our study, VAN monotherapy provided limited protection against relapse consistent with previously reported recurrence patterns (82), whereas the combination therapy (abafungin + VAN) significantly improved survival and supported sustained recovery in body weight. These improved therapeutic outcomes were associated with suppression of key virulence determinants during infection. Mice receiving the combination regimen exhibited markedly reduced fecal spore burdens compared with those treated with VAN alone, indicating decreased spore persistence and a reduced potential for disease relapse. In addition, fecal toxin levels were substantially diminished in the combination-treated group, demonstrating effective *in vivo* suppression of toxin production. Because toxin-mediated epithelial damage is the primary driver of pathology during *C. difficile* infection, attenuation of toxin burden likely contributes to the improved therapeutic outcomes observed. Together, these findings demonstrate that this adjunctive strategy may represent a potential approach for reducing recurrence and improving clinical management of *C. difficile* infection.

The identification of abafungin as a potent anti-*C. difficile* inhibitor is also notable in the context of fungal-bacterial interactions within the gut microbiota. Abafungin possesses fungicidal activity against *Candida* species and other yeasts (26), primarily through membrane disruption and interference with sterol metabolism. The ability of abafungin to target both fungal organisms and *C. difficile* suggests a dual-action approach that may simultaneously limit fungal overgrowth and reduce bacterial virulence reservoirs associated with CDI recurrence. Given that antibiotic-induced fungal blooms have been shown to exacerbate intestinal inflammation and promote CDI in animal models (29–31), agents with combined antifungal and anti-*C. difficile* activity such as abafungin may contribute to restoration of a more balanced intestinal ecosystem and improved disease control.

In conclusion, this study identifies abafungin as a potent anti-*C. difficile* inhibitor. Abafungin demonstrated potent activity against a diverse panel of *C. difficile* clinical isolates, including hypervirulent strains, while exhibiting minimal activity against representative gut commensal bacteria. Beyond its bactericidal activity, abafungin exhibited antivirulence effects by suppressing toxin production and inhibiting spore formation and germination, which are processes that contribute to disease persistence and recurrence. The compound also maintained stable activity under simulated gastrointestinal conditions and displayed a favorable selectivity profile in mammalian cells. Importantly, adjunctive therapy with abafungin enhanced the efficacy of VAN and reduced recurrence in a murine model of CDI. Together, these findings support abafungin as a useful lead scaffold for structure–activity relationship (SAR) optimization, aimed at developing improved therapeutics for CDI.

It is worth noting, however, that this study has several limitations. First, although the combination of abafungin and VAN demonstrated efficacy in a murine model of recurrent CDI, the absence of an abafungin monotherapy arm precluded assessment of its standalone therapeutic efficacy *in vivo* and limited the ability to determine whether abafungin alone has therapeutic efficacy *in vivo* or whether the effect observed is additive or synergistic in the context of vancomycin treatment. Future studies incorporating abafungin monotherapy will be important to address this question. Additionally, further pharmacokinetic and pharmacodynamic studies are needed to define optimal dosing and intestinal exposure. Second, although transcriptomic and magnesium antagonism studies suggest a potential association with metal ion homeostasis pathways, additional mechanistic investigations are required to identify the definitive mechanism, including, but not limited to, intracellular metal quantification, biochemical binding studies, or membrane integrity analyses. Finally, broader microbiome analyses and evaluation in combination with other clinically relevant therapies, including fidaxomicin, will be important to further assess its translational potential. Continued medicinal chemistry and *in vivo* evaluation will be instrumental in advancing abafungin-based candidates toward clinical utility.

## Data Availability

RNA-seq data were deposited in the NCBI Sequence Read Archive (SRA) under accession number PRJNA1401874.

## SUPPLEMENTAL MATERIAL

Supplemental material aac.00598-26-s0001.docx Tables S1 to S5; Fig. S1 and S2.

ASM does not own the copyrights to Supplemental Material that may be linked to, or accessed through, an article. The authors have granted ASM a non-exclusive, world-wide license to publish the Supplemental Material files. Please contact the corresponding author directly for reuse.
