# Supplementary material for: Antibacterial and antivirulence activities of abafungin against Clostridioides difficile: in vitro and in vivo studies
Source: Antimicrob Agents Chemother. 2026 Aug 13;70(9):e00598-26. doi: 10.1128/aac.00598-26 (PMC13713006; doi:10.1128/aac.00598-26)
Supplement: Supplementary file 1 [file aac.00598-26-s0001.docx]

**Supplemental data**

**Antibacterial and Antivirulence Activities of Abafungin Against *Clostridioides difficile*: *in vitro* and *in vivo* Studies**

Ahmed A. Abouelkhair^1,2^, Nader S. Abutaleb^1,3^, Sahar Abdelrazek^1,4^**,** and Mohamed N. Seleem^1,5*^

^1^Department of Biomedical Sciences and Pathobiology, Virginia-Maryland College of Veterinary Medicine, Virginia Polytechnic Institute and State University, Blacksburg, VA 24061, USA.

^2^Department of Bacteriology, Mycology, and Immunology, Faculty of Veterinary Medicine, University of Sadat City, Sadat City 32897, Menoufia, Egypt.

^3^Department of Microbiology and Immunology, Faculty of Pharmacy, Zagazig University, Zagazig, Al-Sharqia 44519, Egypt.

^4^ Department of Plant Pathology, Faculty of Agriculture, Assiut University, Assiut 71515, Egypt.

^5^Center for One Health Research, Virginia Polytechnic Institute and State University, Blacksburg, VA 24061, USA.

*Address correspondence to Mohamed N. Seleem, [seleem@vt.edu](mailto:seleem@vt.edu)

Department of Biomedical Sciences and Pathobiology

Virginia-Maryland College of Veterinary Medicine

Virginia Polytechnic Institute and State University

1410 Prices Fork Rd, Blacksburg, VA, 24061, USA

**Table 1: Full description of the source and the characters of *C. difficile* strains (Thirty-two pathogenic strains):**

| **No.** | ***C.difficile* Strain** | **Alternate designation** | **Source** | **Characters** |
| --- | --- | --- | --- | --- |
| 1 | **NR- 49302** | *C. difficile,* Isolate 20111075 | **BEI:** isolate 20111075was obtained from the stool of an elderly male patient with a healthcare-associated (HA) *C. difficile* infection in Minnesota, USA, in 2010. | *tcdA*, *tcdB* and *tcdC* of the PaLoc operon. This isolate is reported to be negative for the *C. difficile* binary toxin (CDT). |
| 2 | **NR- 49304** | *C. difficile,* Isolate 20120956 | **BEI:** isolate 20120956 was obtained from the stool of an older male patient with a healthcare-associated (HA) *C. difficile* infection in southern USA in 2011. | containing *tcdA*, *tcdB* and *tcdC* of the PaLoc operon. This isolate is reported to be negative for the *C. difficile* binary toxin (CDT). |
| 3 | **NR- 49306** | *C. difficile,* Isolate 20110997 | **BEI:** isolate 20110997 was obtained from the stool of an elderly male patient with a community-associated (CA) *C. difficile* infection in midwestern USA in 2011. | containing *tcdA*, *tcdB* and *tcdC* of the PaLoc operon. This isolate is reported to be negative for the *C. difficile* binary toxin (CDT). |
| 4 | **NR- 49307** | *C. difficile,* Isolate 20120020 | **BEI:** isolate 20120020 was obtained from the stool of an elderly female patient with a community-associated (CA) *C. difficile* infection in northeastern USA, in 2011. | containing *tcdA*, *tcdB* and *tcdC* of the PaLoc operon. This isolate is reported to be negative for the *C. difficile* binary toxin (CDT). |
| 5 | **NR- 49308** | *C. difficile,* Isolate 20120166 | **BEI:** isolate 20120166 was obtained from the stool of an elderly female patient with a community-associated (CA) *C. difficile* infection in Tennessee, USA, in 2011. | containing *tcdA*, *tcdB* and *tcdC* of the PaLoc operon. This isolate is reported to be negative for the *C. difficile* binary toxin (CDT). |
| 6 | **NR- 49310** | *C. difficile,* Isolate 20110986 | **BEI:** isolate 20110986 was obtained from the stool of a young adult male patient with a community-associated (CA) *C. difficile* infection in midwestern USA in 2011. | containing *tcdA*, *tcdB* and *tcdC* (with 39 base pair deletion) of the PaLoc operon as well as the *C. difficile* binary toxin (CDT). |
| 7 | **NR- 49313** | *C. difficile,* Isolate 20110963 | **BEI:** isolate 20110963 was obtained from the stool of an elderly female patient with a healthcare-associated (HA) *C. difficile* infection in Minnesota, USA, in 2011. | containing *tcdA*, *tcdB* and *tcdC* of the PaLoc operon. This isolate is reported to be negative for the *C. difficile* binary toxin (CDT). |
| 8 | **NR- 49314** | *C. difficile,* Isolate 20121412 | **BEI:** isolate 20121412 was obtained from the stool of a young female patient with a community-associated (CA) *C. difficile* infection in Georgia, USA, in 2011. | containing *tcdA*, *tcdB* and *tcdC* of the PaLoc operon. This isolate is reported to be negative for the *C. difficile* binary toxin (CDT). |
| 9 | **NR- 49318** | *C. difficile,* Isolate 20110973 | **BEI:** isolate 20110973 was obtained from the stool of a pediatric female patient with a community-associated (CA) *C. difficile* infection in midwestern USA in 2011. | containing *tcdA*, *tcdB* and *tcdC* of the PaLoc operon. This isolate is reported to be negative for the *C. difficile* binary toxin (CDT). |
| 10 | **NR- 49319** | *C. difficile,* Isolate 20110992 | **BEI:** isolate 20110992 was obtained from the stool of an elderly male patient with a community-associated (CA) *C. difficile* infection in midwestern USA in 2011. | containing *tcdA*, *tcdB* and *tcdC* of the PaLoc operon. This isolate is reported to be negative for the *C. difficile* binary toxin (CDT). |
| 11 | **AR-1067 (ribotype - 027)** | *C. difficile,* Isolate1067 | **CDC:** isolated from Homosapien (Unknown) in 2016, USA (Ribotype -027). | It is positive for *tcdA, tcdB, cdtA, and cdtB* |
| 12 | **AR -1072 (ribotype - 027)** | *C. difficile,* Isolate1072 | **CDC:** isolated from Homosapien (Unknown) in 2016, USA (Ribotype -027). | It is positive for *tcdA, tcdB, cdtA, and cdtB* |
| 13 | **AR -1076 (ribotype - 027)** | *C. difficile,* Isolate1076 | **CDC:** isolated from Homosapien (Unknown) in 2016, USA | It is positive for *tcdA, tcdB,* *cdtA,* and *cdtB* |
| 14 | **AR -1077** | *C. difficile,* Isolate1077 | **CDC:** isolated from Homosapien (Unknown) in 2016, USA | It is positive for *tcdA, tcdB,* *cdtA,* and *cdtB* |
| 15 | **AR -1078** | *C. difficile,* Isolate1078 | **CDC:** isolated from Homosapien (Unknown) in 2016, USA | It is positive for *tcdA, tcdB,* but negative with *cdtA, cdtB* |
| 16 | **AR -1079** | *C. difficile,* Isolate1079 | **CDC:** isolated from Homosapien (Unknown) in 2016, USA | It is positive for *tcdA, tcdB,* but negative with *cdtA, cdtB* |
| 17 | **AR -1082** | *C. difficile,* Isolate1082 | **CDC:** isolated from Homosapien (Unknown) in 2016, USA | It is positive for *tcdA, tcdB,* but negative with *cdtA, cdtB* |
| 18 | **AR -1083** | *C. difficile,* Isolate1083 | **CDC:** isolated from Homosapien (Unknown) in 2016, USA | It is positive for *tcdA, tcdB,* *cdtA,* and *cdtB* |
| 19 | **AR -1085** | *C. difficile,* Isolate1085 | **CDC:** isolated from Homosapien (Unknown) in 2016, USA | It is positive for *tcdA, tcdB,* but negative with *cdtA, cdtB* |
| 20 | **AR -1086** | *C. difficile,* Isolate 1086 | **CDC:** isolated from Homosapien (Unknown) in 2016, USA | It is positive for *tcdA, tcdB,* but negative with *cdtA, cdtB* |
| 21 | **AR -1087** | *C. difficile,* Isolate1087 | **CDC:** isolated from Homosapien (Unknown) in 2016, USA | It is positive for *tcdA, tcdB,* but negative with *cdtA, cdtB* |
| 22 | **AR -1088** | *C. difficile,* Isolate1088 | **CDC:** isolated from Homosapien (Unknown) in 2016, USA | It is positive for *tcdA, tcdB,* but negative with *cdtA, cdtB* |
| 23 | **AR -1089** | *C. difficile,* Isolate1089 | **CDC:** isolated from Homosapien (Unknown) in 2016, USA | It is positive for *tcdA, tcdB,* but negative with *cdtA, cdtB* |
| 24 | **AR -1090** | *C. difficile,* Isolate1090 | **CDC:** isolated from Homosapien (Unknown) in 2016, USA | It is positive for *tcdA, tcdB,* but negative with *cdtA, cdtB* |
| 25 | **AR -1092** | *C. difficile,* Isolate1092 | **CDC:** isolated from Homosapien (Unknown) in 2016, USA | It is positive for *tcdA, tcdB,* *cdtA,* and *cdtB* |
| 26 | **AR -1093** | *C. difficile,* Isolate1093 | **CDC:** isolated from Homosapien (Unknown) in 2016, USA | It is positive for *tcdA, tcdB,* but negative with *cdtA, cdtB* |
| 27 | **AR -1094** | *C. difficile,* Isolate1094 | **CDC:** isolated from Homosapien (Unknown) in 2016, USA | It is positive for *tcdA, tcdB,* *cdtA,* and *cdtB* |
| 28 | **AR -1095 (ribotype - 027)** | *C. difficile,* Isolate1095 | **CDC:** isolated from Homosapien (Unknown) in 2016, USA | It is positive for *tcdA, tcdB,* *cdtA,* and *cdtB* |
| 29 | **AR -1096** | *C. difficile,* Isolate1096 | **CDC:** isolated from Homosapien (Unknown) in 2016, USA | It is positive for *tcdA, tcdB,* but negative with *cdtA, cdtB* |
| 30 | **ATCC 630**  **(Ribotype - 012)** | *C. difficile,* IsolateBAA-1382 | **ATCC:** Switzerland | It is positive for *tcdA, tcdB,* but negative with *cdtB* |
| 31 | **ATCC 43255**  **(ribotype - 087)** | *C. difficile,* Isolate VPI 10463 | **ATCC:** Abdominal wound (Ribotype-087) | It is positive for *tcdA, tcdB,* but negative with *cdtB* |
| 32 | **ATCC BAA-1870 (ribotype - 027)** | *C. difficile,* Isolate4118 | **ATCC:** Clinical isolate (Ribotype-027) | It is positive for *tcdA, tcdB,* and *cdtB* |

**CDC**; The Centers for Disease Control and Prevention.

**BEI Resources**; The Biodefense and Emerging Infections Research Resources Repository.

**ATCC**; The American Type Culture Collection.

**Table 2: Full description of the source and the characters of gut microbiota (Twenty-two strains):**

| **No.** | ***C.difficile* Strain** | **Alternate designation** | **Source** |
| --- | --- | --- | --- |
| 1 | ***Bifidobacteium longum* HM-846** | *Bifidobacterium longum subsp. longum,* 1-6B | Isolated in 2006 from feces of a six-year-old healthy human child in Russia. |
| 2 | ***Bifidobacterium angulatum* HM-1189** | *Bifidobacterium angulatum,* F16_22 | Isolated from human stool in Guelph, Ontario, Canada. |
| 3 | ***Bifidobacterium longum* subsp. *longum***  **HM-847** | *Bifidobacterium longum subsp. longum,* 35B | Isolated from a one-year-old human patient. |
| 4 | ***Bifidobacterium breve* ATCC 15700** | *Bifidobacterium breve*  S1 (Variant a) | Intestine |
| 5 | ***Bifidobacteium longum* HM-845** | *Bifidobacterium longum subsp. longum,* Strain 44B | Isolated from a one-year-old human patient. |
| 6 | ***Bifidobacterium longum subsp. longum,* HM-848** | *Bifidobacterium longum subsp. longum,* 2-2B | Isolated from a six-year-old human patient. |
| 7 | ***Bacteroides finegoldii***  **HM-727** | *Bacteroides finegoldii,* CL09T03C10 | Isolated from healthy adult human feces in Massachusetts, USA. |
| 8 | ***Bacteroides stercoris***  **HM-1036** | *Bacteroides stercoris,* CC31F | Isolated in 2010 from colonic biopsy tissue of a human subject in Victoria, British Columbia, Canada. |
| 9 | ***Bacteroides fragilis***  **HM-711** | *Bacteroides fragilis,* CL05T00C42 | Isolated from healthy adult feces in Massachusetts, USA. |
| 10 | ***Bacteroides fragilis***  **HM-709** | *Bacteroides fragilis,* CL07T00C01 | Isolated from healthy adult feces in Massachusetts, USA. |
| 11 | ***Bacteroides fragilis*,**  **HM-714** | *Bacteroides fragilis,* CL03T12C07 | Isolated from healthy adult human feces in Massachusetts, USA. |
| 12 | ***Lacticaseibacillus rhamnosus* ATCC 53103** |  | Faeces |
| 13 | ***Lacticaseibacillus rhamnosus,***  **HM-106** | *Lacticaseibacillus rhamnosus,* LMS2-1 | *Lacticaseibacillus rhamnosus* (*L. rhamnosus*), strain LMS2-1 is a human gastrointestinal isolate. |
| 14 | ***Lactobacillus gasseri* ATCC 19992** |  |  |
| 15 | ***Lactobacilius casei* ATCC 334** |  | Dairy products |
| 16 | ***Levilactobacillus brevis* ATCC 14869** | *Lactobacillus brevis* Bb14 | Faeces |
| 17 | ***Lactobacillus* HM-100** |  | Faeces |
| 18 | ***Veillonella* sp.,**  **HM-49** | *Veillonella sp*., 6_1_27 | Isolated from rectal biopsy tissue taken from a healthy, 59-year-old male patient undergoing a colon cancer screen procedure in Calgary, Alberta, Canada in 2007. |

**Table 3. MICs (μg/mL) of azole antifungals and reference antibiotics against *C. difficile* strains**

| **No.** | **Compound** | **MIC (μg/ml)** |
| --- | --- | --- |
|  |  | ***C. difficile* ATCC BAA-1870** |
| 1 | **Albaconazole** | >64 |
| 2 | **Fluconazole** | >64 |
| 3 | **Isavuconazole** | >64 |
| 4 | **Itraconazole** | >16 |
| 5 | Posaconazole | >64 |
| 6 | **Ravuconazole** | >64 |
| 7 | **Terconazole** | >64 |
| 8 | **Voriconazole** | >64 |
| 9 | **Efinaconazole** | 64 |
| 10 | **Epoxiconazole** | 64 |
| 11 | **Ketoconazole** | 32 |
| 12 | **Luliconazole** | 32 |
| 13 | **Bifonazole** | 4 |
| 14 | **Clotrimazole** | 2 |
| 15 | **Isoconazole** | 2 |
| 16 | **Sulconazole** | 2 |
| 17 | **Miconazole** | 1 |
| 18 | **Econazole** | 1 |
| 19 | **Tioconazole** | 1 |
| 20 | **Abafungin** | ≤0.5 |
| **Vancomycin** | | 1 |
| **Fidaxomicin** | | ≤0.0078 |

**Table 4. Interactions between Abafungin and vancomycin against *C. difficile* clinical isolates**

| **No.** | ***C.difficile* strain**  **ATCC/BEI designations** | **MIC (μg/ml)** | | | | **ΣFIC index*^a^*** | **Interpretation*^b^*** |
| --- | --- | --- | --- | --- | --- | --- | --- |
|  |  | **VAN** | | **Abafungin** | |  |  |
|  |  | **Alone** | **Combined with abafungin** | **Alone** | **Combined with VAN** |  |  |
| 1 | **AR-1067** | 2 | 1 | 0.5 | 0.25 | 1 | **ADD** |
| 2 | **AR-1072** | 2 | 0.25 | 0.5 | 0.25 | 0.625 | **ADD** |
| 3 | **AR-1086** | 0.5 | 0.25 | 0.5 | 0.125 | 0.75 | **ADD** |
| 4 | **AR-1092** | 2 | 0.5 | 0.5 | 0.25 | 0.75 | **ADD** |
| 5 | **AR-1095** | 2 | 0.5 | 0.5 | 0.125 | 0.5 | **SYN** |
| 6 | **NR-49302** | 0.5 | 0.0078 | 0.5 | 0.25 | 0.5156 | **SYN** |
| 7 | **NR-49304** | 1 | 0.25 | 0.25 | 0.125 | 0.75 | **ADD** |
| 8 | **NR-49318** | 0.5 | 0.06 | 0.5 | 0.125 | 0.37 | **SYN** |
| 9 | **NR-49319** | 0.5 | 0.25 | 0.5 | 0.03 | 0.56 | **SYN** |
| 10 | **ATCC 43255** | 0.5 | 0.0078 | 0.125 | 0.06 | 0.4956 | **SYN** |
| 11 | **ATCC BAA -1870** | 1 | 0.0078 | 0.5 | 0.125 | 0.2578 | **SYN** |

^a^ΣFIC, fractional inhibitory concentration summation.

^b^An ΣFIC index of ≤0.5 is considered synergistic (SYN); an ΣFIC index of >0.5 to 1.25 is considered additive (ADD); an ΣFIC index of >1.25 to 4 is considered indifferent (IND); and an ΣFIC index of >4 is considered antagonistic.

**Table 5. Interactions between Abafungin and metronidazole against *C. difficile* clinical isolates**

| **No.** | ***C.difficile* strain**  **ATCC/BEI designations** | **MIC (μg/ml)** | | | | **ΣFIC index*^a^*** | **Interpretation*^b^*** |
| --- | --- | --- | --- | --- | --- | --- | --- |
|  |  | **MTZ** | | **Abafungin** | |  |  |
|  |  | **Alone** | **Combined with abafungin** | **Alone** | **Combined with MTZ** |  |  |
| 1 | **AR-1067** | 0.25 | 0.03 | 0.5 | 0.25 | 0.62 | **ADD** |
| 2 | **AR-1072** | 0.25 | 0.06 | 0.5 | 0.25 | 0.74 | **ADD** |
| 3 | **AR-1086** | 0.25 | 0.03 | 0.5 | 0.25 | 0.62 | **ADD** |
| 4 | **AR-1092** | 0.25 | 0.25 | 0.5 | 0.25 | 1.5 | **IND** |
| 5 | **AR-1095** | 0.25 | 0.06 | 0.25 | 0.125 | 0.74 | **ADD** |
| 6 | **NR-49302** | 0.5 | 0.03 | 0.5 | 0.25 | 0.56 | **SYN** |
| 7 | **NR-49304** | 0.5 | 0.125 | 0.25 | 0.06 | 0.49 | **SYN** |
| 8 | **NR-49318** | 0.25 | 0.06 | 0.5 | 0.03 | 0.3 | **SYN** |
| 9 | **NR-49319** | 0.25 | 0.03 | 0.5 | 0.25 | 0.62 | **ADD** |
| 10 | **ATCC 43255** | 0.25 | 0.06 | 0.125 | 0.06 | 0.72 | **ADD** |
| 11 | **ATCC BAA-1870** | 0.125 | 0.03 | 0.5 | 0.125 | 0.49 | **SYN** |

^a^ΣFIC, fractional inhibitory concentration summation.

^b^An ΣFIC index of ≤0.5 is considered synergistic (SYN); an ΣFIC index of >0.5 to 1.25 is considered additive (ADD); an ΣFIC index of >1.25 to 4 is considered indifferent (IND); and an ΣFIC index of >4 is considered antagonistic.

**Figure S1.** Total count of *C. difficile* ATCC 43255 after being treated with Abafungin, Van, FDX, MTZ, and DMSO at 0.25× MIC. Treatment with any of the tested agents at 0.25× MIC did not affect bacterial counts relative to the DMSO control.

**Figure S2. Global transcriptomic response of *C. difficile* ATCC 630 to abafungin and vancomycin exposure. (A)** Principal component analysis (PCA) demonstrating separation of transcriptomic profiles between abafungin-treated samples and DMSO-treated control samples. **(B)** Hierarchical clustering heatmap of the top 50 most differentially expressed genes across treatment groups. Expression values were normalized and displayed as Z-scores to illustrate relative changes in gene expression patterns between conditions. Color intensity represents relative expression levels (scaled log₂ expression values), with red indicating upregulation and blue indicating downregulation. Distinct clustering patterns demonstrate consistent transcriptional responses to abafungin treatment. **A, abafungin-treated samples; C, DMSO-treated control samples.**
